# Pre-Pregnancy Obesity vs. Other Risk Factors in Probability Models of Preeclampsia and Gestational Hypertension

**DOI:** 10.3390/nu12092681

**Published:** 2020-09-02

**Authors:** Małgorzata Lewandowska, Barbara Więckowska, Stefan Sajdak, Jan Lubiński

**Affiliations:** 1 Medical Faculty, Lazarski University, 02-662 Warsaw, Poland; 2 Division of Gynecological Surgery, University Hospital, 33 Polna Str., 60-535 Poznan, Poland; ssajdak@ump.edu.pl; 3 Department of Computer Science and Statistics, Poznan University of Medical Sciences, 60-806 Poznan, Poland; barbara.wieckowska@ump.edu.pl; 4 Department of Genetics and Pathology, International Hereditary Cancer Center, Pomeranian Medical University, 71-252 Szczecin, Poland; lubinski@pum.edu.pl

**Keywords:** obesity, gestational hypertension, preeclampsia, pregnancy, risk factors, prediction

## Abstract

In the face of the obesity epidemic around the world, attention should be focused on the role of maternal obesity in the development of pregnancy. The purpose of this analysis was to evaluate the prediction of preeclampsia (PE) and isolated gestational hypertension (GH) for a number of maternal factors, in order to investigate the importance of pre-pregnancy obesity (body mass index, BMI ≥ 30 kg/m^2^), compared to other risk factors (e.g., prior PE, pregnancy weight gain (GWG), infertility treatment, interpregnancy interval, family history, the lack of vitamin supplementation, urogenital infection, and socioeconomic factors). In total, 912 women without chronic diseases were examined in a Polish prospective cohort of women with a singleton pregnancy (recruited in 2015–2016). Separate analyses were performed for the women who developed GH (*n* = 113) vs. 775 women who remained normotensive, as well as for those who developed PE (*n* = 24) vs. 775 controls. The probability of each disease was assessed for the base prediction model (age + primiparity) and for the model extended by one (test) variable, using logistic regression. Three measures were used to assess the prediction: area under curve (AUC) of the base and extended model, integrated discrimination improvement (IDI) (the index shows the difference between the value of the mean change in the predicted probability between the group of sick and healthy women when a new factor is added to the model), and net reclassification improvement (NRI) (the index focuses on the reclassification table describing the number of women in whom an upward or downward shift in the disease probability value occurred after a new factor had been added, including results for healthy and sick women). In the GH prediction, AUC increased most strongly when we added BMI (kg/m^2^) as a continuous variable (AUC = 0.716, *p* < 0.001) to the base model. The highest IDI index was obtained for prior GH/PE (IDI = 0.068, *p* < 0.001). The addition of BMI as a continuous variable or BMI ≥ 25 kg/m^2^ improved the classification for healthy and sick women the most (NRI = 0.571, *p* < 0.001). In the PE prediction, AUC increased most strongly when we added BMI categories (AUC = 0.726, *p* < 0.001) to the base model. The highest IDI index was obtained for prior GH/PE (IDI = 0.050, *p* = 0.080). The addition of BMI categories improved the classification for healthy and sick women the most (NRI = 0.688; *p* = 0.001). After summing up the results of three indexes, the probability of hypertension in pregnancy was most strongly improved by BMI, including BMI ≥ 25 kg/m^2^ for the GH prediction, and BMI ≥ 30 kg/m^2^ for the PE prediction. Main conclusions: Pre-pregnancy BMI was the most likely factor to increase the probability of developing hypertension in pregnancy, compared to other risk factors. Hierarchies of PE and GH risk factors may suggest different (or common) mechanisms of their development.

## 1. Introduction

The severity of the obesity epidemic in the world is one of the reasons why we focused our attention on the assessment of the role of maternal obesity in the development of pregnancy [1,2]. Many studies have confirmed that pre-pregnancy obesity is an important part of the Development of Health and Disease (DOHaD) concept, in which factors influencing the intrauterine environment are risk factors for non-communicable diseases in adulthood. The search for evidence linking pre-pregnancy obesity to fetal “programming” and intergenerational effects is ongoing [3,4,5,6].

Pre-pregnancy obesity has been recognized as a strong risk factor for pregnancy-induced hypertension, independent of the influence of other confounders [7,8,9,10,11,12], but this does not prejudge us against the importance of obesity compared to other maternal risk factors [7]. The hierarchy of risk factors in predicting hypertension in pregnancy has not been established, and choosing how to compare their effects can be challenging.

Pregnancy-induced hypertension (PIH) is one of the serious complications of pregnancy and includes two main forms, preeclampsia (PE) (a more severe form which is phenotypically heterogeneous and accompanied by organ dysfunction) and isolated gestational hypertension (GH) [13,14,15]. Pregnancy-induced hypertension occurs, on average, in 10% of the pregnant population, but, in some regions of the world, its incidence is higher [14,15]. Hypertension in pregnancy, especially preeclampsia (PE), is one of the main causes of mortality and morbidity in mothers and their babies, and the effects of the disease are not only limited to the perinatal period, but also have long-term effects related to, e.g., a future higher risk of cardiovascular disease in both mothers and babies [13,15,16,17,18,19]. Early identification of women at risk of PIH is needed so they can be placed under increased surveillance as early as possible.

However, despite significant advances in understanding many of the molecular processes involved in the pathogenesis of preeclampsia, a reliable method of predicting hypertension in pregnancy remains yet to be developed. Maternal risk factors are still an important element in risk identification [13,15,20]. In many studies, a higher risk of preeclampsia (PE) and/or GH has been associated with pre-pregnancy obesity (body mass index, BMI ≥ 30 kg/m^2^), comorbidities (such as chronic hypertension), older maternal age, primiparity, diagnosis of PE in a previous pregnancy, as well as with PE in the mother or a sister, and short or long gaps between pregnancies. Other factors include assisted reproductive techniques, race, and ethnicity (higher risk can be observed in Afro-Caribbean and South Asian women), as well as excess weight gain in pregnancy (GWG) [13,15,20].

Various scientific societies list pre-pregnancy obesity among the factors of moderate or high risk of preeclampsia. However, it is not yet established how obesity affects the various phenotypes of hypertension. Some studies have linked increases in BMI to the development of late-onset PE, but not to early-onset PE; however, other studies have not confirmed these results [21]. The availability in the literature of a much larger number of studies which only deal with women developing preeclampsia (PE), as compared to studies covering isolated gestational hypertension (GH), makes it difficult to identify the differences between PE and GH. Both of these forms of hypertension in pregnancy require research, as both are associated with poorer neonatal outcomes, as compared to the pregnancy outcomes of normotensive women [7,13,18,22].

The results based on the calculation of the odds ratios and/or predictive indicators available in the literature have their drawbacks. In the case of the odds ratio, a disadvantage is the inability to compare this measure between different variables, e.g., between continuous variables expressed in different units. Area under curve (AUC) in multivariate regression models can be compared for any variable; however, AUC has no good clinical interpretation. In addition, assessing markers with these measures omits those with missing data, leading to results for slightly different groups. All this makes it difficult to compare the hierarchy of factors. Many studies have shown that adding more markers to the predictive model improves the quality of the prediction or classification to the ill and the healthy group of patients. However, this does not explain which factor in the multivariate model is the strongest.

Our aim was to investigate in a joint cohort how important role pre-pregnancy obesity of the mother plays in the likelihood of developing isolated gestational hypertension (GH) and preeclampsia (PE), compared to the role of other risk factors. Our analysis was based on the measurement of several statistical indicators, in the evaluation of multivariate probability models. In order to broaden the assessment of the quality of the prediction of potential GH and PE risk markers, in addition to AUC, we used two newer coefficients: Integrated Discrimination Improvement (IDI) and Net Reclassification Improvement (NRI). The IDI measures the mean change in disease probability when a new marker is added to the model. The NRI, on the other hand, provides a clinically very favorable interpretation by calculating the percentage of persons in whom the addition of the marker under examination improves or worsens the prediction (classification). Missing data were treated as an additional category, and each analysis was based on the same dataset [23,24,25]. We have not found a similar study in the literature.

We studied a cohort of pregnant women prospectively recruited during the first trimester, when pregnancy outcomes were unknown.

## 2. Materials and Methods

The study was conducted in accordance with the Helsinki Declaration. It was registered and approved by the Bioethics Committee in Poznań, Poland (Medical University No. 769/15). All the women recruited for this study expressed their informed consent before completing the survey questionnaire.

### 2.1. Participants and Methods

The current analysis is based on the data from a prospectively recruited cohort of pregnant women. The participants were recruited in 2015–2016. The research center was the Gynecology and Obstetrics Clinical Hospital of the Medical University of Poznań, Poland. This center is a third-level hospital in the field of obstetrics.

The recruitment process took place at the central laboratory of this clinical hospital. Information about the study was displayed in a prominent place and was available to any woman who voluntarily reported for laboratory tests. The eligibility criteria included the following: (1) being a Caucasian woman from the Wielkopolska region, which ruled out the influence of ethnic differences (an important risk factor for many diseases), and matched the study groups in terms of the quality of prenatal care; (2) aged 18–45 years at conception; (3) with a singleton pregnancy; (4) gestational age of 10–14 weeks; (5) the absence of chronic diseases, especially hypertension and diabetes mellitus, as well as kidney and/or liver dysfunction, and the absence of any immunological and inflammatory diseases and thromboembolism; and (6) the absence of fetal defects and the birth of a (phenotypically) normal child of ≥25th week of gestation.

The recruitment was the first stage of the study. The participants completed the research questionnaire (they did it on their own, in the presence of a midwife). The questionnaire was used to collect the information about the current pregnancy and previous pregnancies, accompanying symptoms, supplementation with folic acid, and other multivitamin preparations (typically recommended for pregnant women), as well as the use of other drugs and stimulants (the women informed us about their smoking before pregnancy and at the time of recruitment, and no participants reported alcohol consumption during pregnancy). Sociodemographic data were recorded, as well. The questionnaire also included questions about family health history (including chronic hypertension). The questionnaire also asked about the consumption of several groups of food products (number of servings per week), but it was not a standardized nutritional questionnaire, and its main purpose was to identify the women using special diets; all the participants reported a mixed diet during the first trimester of pregnancy, and several women reported having followed a vegetarian diet in the past.

In the second stage, after delivery, information was collected on the course of pregnancy, possible complications in the mother, and the results of the newborn. The data were taken from medical records. A change in diet was observed in the women with diagnosed pregnancy-induced hypertension or gestational diabetes (women with hypertension used a low-sodium diet).

All 1300 women who expressed their willingness to participate in the study and met the criteria were invited to take part. Forth-eight women were excluded due to miscarriage <20th week, delivery <25th week, diagnosis of the child’s defect, severe infection during pregnancy, thromboembolism, arterial hypertension before the 20th week, diabetes before the 18th week, and lack of cooperation. In total, 340 women were also excluded due to their incomplete data.

The primary cohort for which the data were analyzed consisted of 912 participants. In this analysis, we examined women who developed pregnancy-induced hypertension during pregnancy, including preeclampsia, PE (*n* = 24) and isolated gestational hypertension, GH (*n* = 113), and those who remained normotensive (*n* = 775).

In the current analysis, the relationships between clinical factors and the development of each of the forms of pregnancy-induced hypertension, preeclampsia, and gestational hypertension were investigated.

### 2.2. Definitions

The definition of pregnancy-induced hypertension was adopted according to the guidelines of national and European scientific societies as systolic and diastolic blood pressure ≥140 mmHg and ≥90 mmHg (respectively) obtained in at least two measurements 4 h apart, developing after the 20th week of gestation in women without previous symptoms of hypertension (measured with an oscilometric device in a sitting position) [13]. Preeclampsia (PE) was diagnosed when hypertension was accompanied by de novo development of one organ system disorder (such as renal dysfunction, hepatic dysfunction, thrombocytopenia, cerebral or visual symptoms, and/or pulmonary edema). In this cohort only proteinuria was found in all the PE cases (≥0.3 g/L) [26]. Gestational hypertension (GH) was diagnosed when only hypertension was detected. Blood pressure (systolic and diastolic) before pregnancy was self-reported. Blood pressure was measured in hospital, and values after childbirth (measured on the postpartum ward) were taken for this analysis.

In gestation week 24–28th, an oral glucose tolerance test (2 h test, for 75 g of glucose) was performed to diagnose gestational diabetes mellitus (GDM). GDM-1 was diagnosed when dietary treatment was sufficient to balance the plasma sugar level. GDM-2 diabetes was diagnosed when the normalization of glycaemia required treatment with insulin.

Gestational age was determined using ultrasound examination (USG). Information on the diagnosis of intrauterine growth restriction (IUGR) was found in medical records, and the diagnosis was based on ultrasound examination during pregnancy. Newborn weight was measured conventionally (in grams) immediately after childbirth using an automatic device. Birth weight percentiles were estimated for gender and gestational age in the study population (based on percentile grids).

### 2.3. Potential Predictors, Independent Variables

In the present analysis, we examined a number of clinical factors identified in the literature as potential risk factors for pregnancy-induced hypertension in order to compare them with the results obtained for the pre-pregnancy body mass index (BMI) [13].

Maternal age (defined as completed age in years at conception) was assessed as a continuous and categorical (c) (category 18–24, 25–29, 30–34, 35–39 and ≥40 years).

The number of previous deliveries was assessed as a categorical variable (c) (0, 1, 2, ≥3 deliveries). Primiparity was assessed as a dichotomous variable (primiparity vs. multiparity).

Interpregnancy interval was assessed as a categorical variable (c): primigravida women, 1, 2, 3–5, 6–10, ≥11 years, and the category “unknown” (cases of missing data).

Pre-pregnancy BMI was calculated as the quotient of pre-pregnancy weight in kg (self-reported) and height in meters (from medical records) squared. BMI was assessed as: a continuous variable (kg/m^2^), or a categorical variable (c) (with the following categories: <18.5, 18.5–24.9, 25.0–29.9 and ≥30 kg/m^2^ for underweight, normal weight, overweight and obesity, respectively), or dichotomous variables (≥25 kg/m^2^ vs. <25 kg/m^2^, and ≥30 kg/m^2^ vs. <30 kg/m^2^).

Height (cm) and pre-pregnancy weight (kg) were assessed as continuous variables.

Gestational weight gain (GWG) was calculated as the difference between the weight before delivery (from medical records) and the pre-pregnancy weight (self-reported). GWG was assessed as a continuous variable (kg) and categorical variable (c), according to the 2009 Institute of Medicine (IOM) recommendations: above, below, and in the range [7]. GWG was also assessed as a dichotomous variables: GWB above the range vs. others; GWG > 10 kg vs. <10 kg; and GWG > 15 kg vs. <15 kg).

Prior GH/PE, infertility treatment, in vitro fertilization (IVF), hypothyroidism, no supplementation with folic acid in 1st trimester (self-reported), no supplementation with multivitamin (multivitamin and multi-microelement) preparations in 2–3 trimester, urogenital infection (in 2–3 trimester and smoking in I trimester, were assessed as dichotomous variables (“yes” vs. “others”).

Family history of (chronic) hypertension was assessed as a categorical (c) variable including paternal and/or maternal hypertension, hypertension in the family but not in the father/mother, and no hypertension in the family. Hypertension in the mother was also assessed as a categorial (c) variable including maternal hypertension, hypertension in the family but not in the mother, and no hypertension in the family. Moreover, hypertension in the father was assessed as a categorial (c) variable including paternal hypertension, hypertension in family but not in the father, and no hypertension in the family.

Education was assessed as a dichotomous variable: education of <12 years (primary and vocational) vs. other (secondary and tertiary education, and the category “unknown” for no data).

Financial status was assessed as a dichotomous variable. The categories were grades on the 5-point Likert scale, given to answers to the question “is your household’s financial status sufficient for your needs” (answer, 1 “definitely No”; answer, 2 “rather No”; answer 3, “hard to say”; answer, 4 “rather Yes”; answer, 5 “definitely Yes”, and category “unknown” for no data). The dichotomous analysis included: lower financial status (answers 1 + 2 + 3) vs. answers 4 + 5, and the category “unknown” for no data.

Place of residence was assessed as a categorical variable (c): village, small town <50,000 inhabitants, and large city >50,000 inhabitants.

### 2.4. Statistical Analyses

The investigated independent variables (potential predictors) were described using mean values and standard deviation (SD) and medians (for continuous variables) or by number and percentage (for categorical variables). The normal distribution of continuous variables was assessed with the Shapiro-Wilk test. Comparisons of the independent variables between the control (normotensive) group and the case group (GH or PE) were performed using the Mann-Whitney test (for comparing continuous variables) and the Chi-squared test or Fisher exact test (for comparing categorical variables); statistical significance (*p*-value) is given for each analysis.

Risk factors were assessed as categorical (c) and/or dichotomous and/or continuous variables (with the measurement units including year, kg, kg/m^2^ and cm). For some risk factors, all three forms of variables were assessed. For missing data in categorical variables, a special category labeled “unknown” was created, allowing us to examine the participants with missing data and evaluate all the variables on the full set of 912 participants.

The analysis of categories (c) can be advantageous because categories do not assume any shape of dependence, there is only a division into categories. When examining categories (c), the shape of the relationship will be discovered in the analysis and used for prediction. This is especially important in some situations, where both lower and higher categories/values increase the odds ratios of the disease.

To achieve the main aim of the study, this analysis assessed the degree of influence of each of the maternal factors tested on GH or PE prediction. All the analyses were performed based on logistic regression models. First, crude odds ratios (ORs) for the disease (GH or PE) incidence (along with 95% confidence intervals) were determined for each maternal factor (independent variable) by means of unidimensional logistic regression; the statistical significance of OR was reported, as determined by Wald’s test (*p*-value).

Subsequently, multi-factor predictive regression models (separately for GH and PE) were built. A small basic regression model was built, which included age and primiparity. Subsequent models were extended, and one additional (tested) variable was added to the base model. Three prediction indexes were used to assess the improvement in prediction (change in disease probability) in the subsequent extended multivariate models (compared to the base model): Integrated Discrimination Improvement (IDI), Net Reclassification Improvement (NRI), and area under receiver operating characteristic curve (AUC under ROC curve) of the basic and extended model. For each of the three indicators, 95% confidence intervals were calculated, and their statistical significance (*p*-value) was checked. High and statistically significant values obtained for the difference of AUC and for IDI and NRI prove good predictive ability of the variable added to the basic regression model [23,24,25].

AUC is a known prediction factor in regression models; the greater the difference between the AUC of the extended model and the AUC of the base model, the greater the improvement in the prediction when a new variable is added to the model. The IDI index shows the difference between the value of the mean change in the predicted probability between the group of sick women and the group of healthy women. The NRI index focuses on the reclassification table describing the number of women in whom an upward or downward shift in the disease probability value occurred after a new factor had been added to the model.

The final stage of the analysis was to determine the order (hierarchy) of the examined maternal factors for each of these measures separately (AUC of the extended model, as well as IDI and NRI) assigning the first place to the variable with the highest measure and the last place to the variable with the lowest measure. Finally, the sum of the sequences obtained from these three measures was calculated, and a new order of significance of the maternal factors was given, highlighting those factors which showed the greatest improvement in prediction in the analyses conducted (ranking first in the sequences).

The *p*-value in each analysis was considered statistically significant when its value was less than 0.05. The analyses were performed by using PQstat v1.8.0 (PQstat Software, Poznań, Poland) software.

## 3. Results

The whole group consisted of 912 women with a singleton pregnancy who had no chronic diseases. In the entire cohort (Supplementary Materials Table S1), there were 271 (29.7%) women with a BMI ≥25 kg/m^2^), among them 98 (10.8%) obese women, including obesity I, II, and III (7.7%, 2.7%, and 0.4%, respectively). The percentage of women with GWG above the IOM recommendation range was 36.8%, but for the women with excessive pre-pregnancy BMI, it was 59.0%, compared to 28.7% for the women with normal BMI (Supplementary Materials Table S1).

Table 1 and Supplementary Materials Table S2 present the characteristics of the control and case group. Compared to normotensive women, the women who developed hypertension during pregnancy (gestational hypertension (GH) or preeclampsia (PE)) had higher mean pre-pregnancy BMI, were older, were more often primiparas, more frequently they reported GH/PE in previous pregnancies, were more often treated due to infertility, had a higher mean interpregnancy interval, smoked more often, more frequently displayed lower socioeconomic indicators, and more often reported a family history of chronic hypertension (in the mother or father).

Importantly, cases (of GH and PE) statistically resulted in significantly worse pregnancy outcomes (lower gestational age of newborns, lower birth weight, and more cases of intrauterine growth restriction (IUGR)) than in normotensive women, but unfavorable pregnancy outcomes in women with PE were more strongly expressed. Preterm delivery <37th and <34th week was significantly more frequent in the women with hypertension, especially in PE cases (54.2% and 25.0%, respectively), compared to normotensive women (5.3% and 1.3%, respectively). GDM occurred more frequently in GH and PE cases, as compared to controls, but the difference was statistically insignificant. Fetal sex was not associated with cases of hypertension.

Table 2 and Supplementary Materials Table S3 show the odds ratios of gestational hypertension (GH) and preeclampsia (PE) for many maternal characteristics. An increase in BMI by 1 kg/m^2^ resulted in a statistically significant increase in GH odds ratios by 16% and an increase in PE odds ratios by 15%.

Among the categorical variables, the GH odds ratios were the highest for the following (in the order of importance): prior GH/PE, pre-pregnancy BMI ≥ 30 kg/m^2^, smoking in first trimester, maternal age ≥40 years, lower education, and others.

A different hierarchy was found in the PE study. The PE odds ratios were the highest for the following (in the order of importance): GH/PE, pre-pregnancy BMI ≥ 30 kg/m^2^, interpregnancy interval, education <12 years, family history with chronic hypertension in the mother, and others. The effect of gestational diabetes, lower maternal height, and fetal sex was insignificant.

The similar profiles were found after being adjusted for maternal age and primiparity (AOR-a), and pre-pregnancy BMI (AOR-b) (Supplementary Materials Table S3).

Table 3 presents AUC values for predicting GH and PE calculated for continuous variables and for the basic multi-factor predictive model (maternal age + primiparity). In the GH and PE study, the AUC of the pre-pregnancy BMI model was higher (and more statistically significant) when compared to the other model AUC.

Table 4 and Table 5 show the results for three prediction indexes (AUC, IDI, and NRI) in predicting GH or PE after the extension of the underlying predictive model (maternal age + primiparity). The indexes, confidence intervals, and *p*-value, as well as the percentage of patients reclassified upward and downward, are presented in Supplementary Materials Tables S4–S6.

Table 4 shows the results for the gestational hypertension (GH) prediction. All AUC values of the extended models were statistically significant (*p* < 0.001), and the *p*-value is presented for comparing the AUC of the extended models with the AUC of the base model. The area under the curve increased most strongly when we added BMI (kg/m^2^) as a continuous variable (AUC = 0.716) to the base model (AUC = 0.600). Other forms of pre-pregnancy BMI are also high in the table, including the BMI category ≥ 25 kg/m^2^ (AUC = 0.685). They are followed by BMI ≥ 30 kg/m^2^ and prior GH/PE, as well as GWG expressed in categories (c) (identical to the result for GWG above the IOM recommendations).

Similarly, the BMI index showed a high predictive value also in the NRI examination. NRI = 0.571 was obtained for both BMI examined as a continuous variable and BMI ≥ 25 kg/m^2^. At the same time, it should be noted that adding these variables to the model improves the quality of classification to a greater extent for healthy women NRI (0) = 49.2% than for the sick ones NRI (1) = 8% (complete results in Supplementary Materials Table S5). The IDI index showed a similar hierarchy of maternal factors, but the highest value of the IDI index was obtained for prior GH/PE (IDI = 0.068) (Supplementary Materials Table S6).

Table 5 shows the results for predicting preeclampsia (PE). The AUC values of the extended models were statistically significant after adding the variables listed in the first half of the table to the prior GH/PE variable. The table presents the *p*-value for comparing the AUC of the extended models to the AUC of the base model. The area under the curve increased most strongly when we added BMI as a categorical variable (c) (AUC = 0.726) or family history with hypertension in the mother (c) (AUC = 0.717) to the base model (AUC = 539). Other forms of pre-pregnancy BMI are also high in the table, with BMI ≥ 30 kg/m^2^ (AUC = 0.703) ranked highest among the categories. The following AUC values in the table were obtained after extending the model with BMI ≥ 25 kg/m^2^, with BMI as a continuous variable and hypertension in the father (c).

Adding BMI to the prediction model, as well as adding family history with hypertension in the mother (c), also allowed us to obtain high NRI coefficients, which, as can be seen in a more detailed analysis of these coefficients, resulted mainly from the improvement of the prediction for the healthy women (positive and high NRI (0)), with a slight worsening of the prediction for the sick women (low and negative NRI (1)) (Supplementary Materials Table S5). The IDI index showed a similar hierarchy of maternal factors, but the highest value of the IDI index was obtained for prior GH/PE (IDI = 0.050) (Supplementary Materials Table S6).

Supplementary Materials Figure S1 shows the strongest NRI and IDI indexes in GH and PE prediction for the models extended with the abovementioned maternal characteristics.

**Figure 1:**
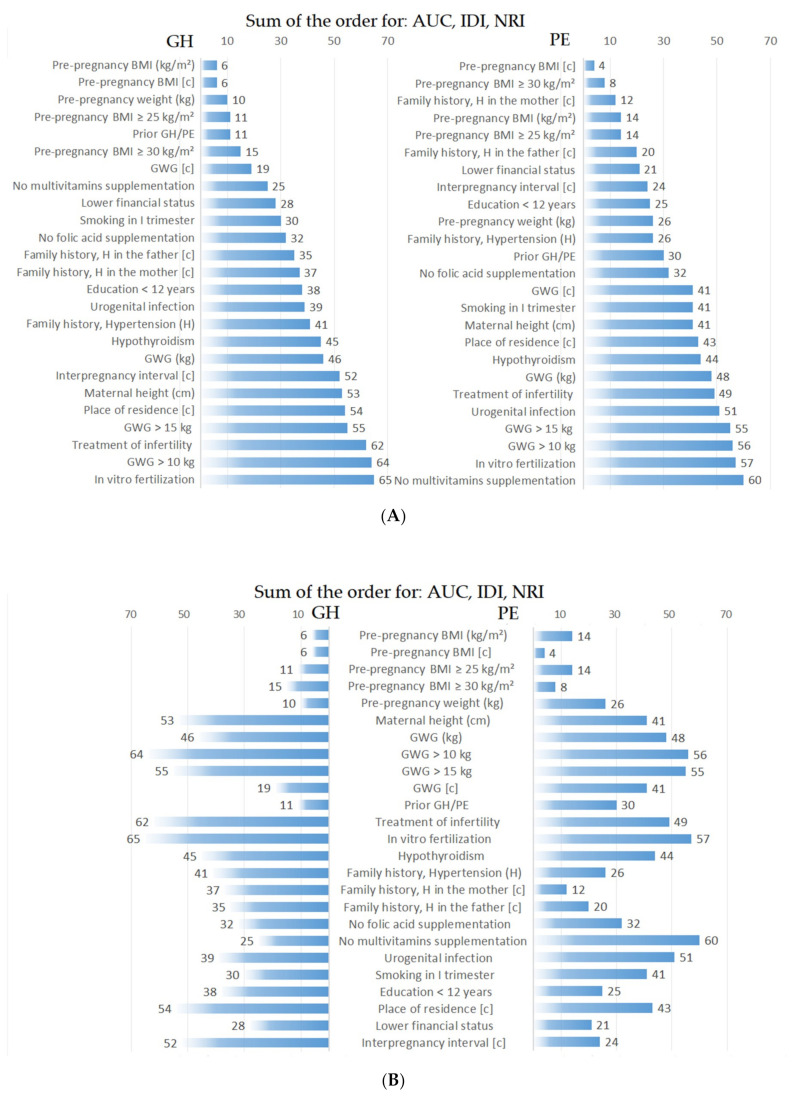
A new order of significance of the maternal factors after the sum of the sequences obtained from the three measures (AUC, IDI and NRI). The image at the top (**A**) highlights those maternal traits that improved predicting the most. The image at the bottom (**B**) shows the variables and their groups and highlights the differences between GH and PE for each variable. BMI, body mass index; (c), categories as described for independent variables in the Methodology; GWG, gestational weight gain; H, hypertension in family history; AUC, area under receiver operating characteristic curve; IDI, Integrated Discrimination Improvement; NRI, Net Reclassification Improvement; GH, gestational hypertension; PE, preeclampsia.

Finally, (see Figure 1) the sum of the sequences obtained from these three measures (AUC, IDI, and NRI) was calculated, resulting in a new order of significance of the maternal factors, highlighting those factors which showed the greatest improvement in prediction in the analyses conducted (ranking first in the sequences). Figure 1A shows that the variables that best improved the GH prediction were pre-pregnancy BMI categories (c), BMI continuous and BMI ≥ 25 kg/m^2^, then prior GH/PE, followed by BMI ≥ 30 kg/m^2^, then GWG categories (c), the lack of multivitamins supplementation in II-III trimester, a lower financial status, chronic hypertension in the father, (c) and then maternal hypertension (c). The variables that best improved the PE prediction were, in turn, pre-pregnancy BMI categories (c) and BMI ≥ 30 kg/m^2^, then chronic hypertension in the mother, then BMI ≥ 25 kg/m^2^, followed by chronic hypertension in the father, lower financial status, interpregnancy interval categories (c), and then education <12 years.

## 4. Discussion

In this analysis, we assessed a prospectively recruited cohort of women with a singleton pregnancy. We examined the women who developed isolated gestational hypertension (GH) (12.4%) and preeclampsia (PE) (2.6%) and those who remained normotensive (85%). The aim of our study was to prioritize the importance of maternal factors in predicting GH and PE. The final result included the results of three prediction measures (AUC of the extended model, IDI, and NRI) that can be used to assess the change (improvement) of the prediction. We found that pre-pregnancy BMI (assessed as a categorical and/or continuous and/or dichotomous variable) was more important in the development of GH and PE compared to other clinical factors.

Importantly, we assessed multivariate prediction models to investigate the importance of pre-pregnancy obesity/overweight, and not to confirm that the greater number of predictors increases the prediction. Our analysis has several advantages over the widely used odds ratio calculations or AUC analysis. IDI determines the mean change in disease probability due to the addition of a new potential marker to the model. NRI gives a clinically very favorable interpretation by providing the percentage of people in whom the addition of the marker under study improves or worsens the prediction (classification). By summing up the marker hierarchy obtained in the AUC, IDI, and NRI examination, we established the final predictor hierarchy taking into consideration many mathematical results.

After taking into account all the prediction measures (AUC, IDI, and NRI), the variables that best improved the GH prediction were pre-pregnancy BMI (including BMI ≥ 25 kg/m^2^), then prior GH/PE, then BMI ≥ 30 kg/m^2^, GWG categories (c), the lack of multivitamins supplementation in the second/third trimester, and a lower financial status. Chronic hypertension in the father was ranked next, followed by maternal hypertension, while infertility treatment and IVF were the last items in the ranking. The variables that best improved the PE prediction were, in turn, pre-pregnancy BMI (including BMI ≥ 30 kg/m^2^), then family history and chronic hypertension in the mother, and then BMI ≥ 25 kg/m^2^, chronic hypertension in the father, lower financial status, interpregnancy interval categories (c), and education <12 years.

Our results showed the great importance of pre-pregnancy obesity/overweight in predicting GH and PE, as reflected in the literature. Many studies have shown that pre-pregnancy obesity is an independent risk factor for hypertension in pregnancy [7,8,9,10,11,12]. Our previous analysis showed that obese women had a statistically significant five-fold higher risk of GH and a nine-fold higher risk of PE compared to women with normal BMI, and these results were obtained after adjusting for many confounding variables, including excessive GWG [7].

Importantly, in the current analysis, there were also differences in the results for BMI; we found the relationship between GH and pre-pregnancy BMI ≥ 25 kg/m^2^, and the relationship between PE and BMI ≥ 30 kg/m^2^. It indicates a lower pre-pregnancy BMI threshold in the GH development, compared to the PE development, which we also found in our previous study; the threshold BMI for the higher risk of GH was 24.3 kg/m^2^ and for the higher risk of PE was 28.2 kg/m^2^ (second cutoff point in the graph) [7]. The differences in these results for GH and PE are small, but they may suggest that severe obesity-related disorders are of greater importance in the development of PE. Early PE (onset <34th week) is characterized by a more severe course, more frequent complications in the mother, and complications in the fetus (intrauterine growth restriction (IUGR), as well as fetal hypoxia or death of the fetus), which may suggest a significant contribution of placental circulation pathology. In late PE, the placenta is often normal; therefore, this form of the disease may be characterized by a lower frequency of fetal complications [13,16,27,28,29]. It was also found that PE manifesting mainly maternal symptoms may display risk factors similar to those of GH (obesity, insulin-resistance, hyperlipidemia, and chronic hypertension), which may suggest the presence of disorders in the mother’s blood vessels before pregnancy [13]. Very interesting results were obtained in a large population study by French authors; higher BMI values were associated with late-onset PE, and not with early onset PE. At the same time, in rich countries, 90% of PE cases have been reported to be of late onset, and low-income countries estimate this percentage at 60–70% [21]. A comparison of the current and older literature shows that, in the middle of the last century, overweight and/or obesity were not mentioned as risk factors for hypertension in pregnancy, but there was no obesity epidemic in those years.

The clinical implications of our result are very direct and clearly related to the need to intensify education in women of childbearing age on the dangers of obesity and overweight, the positive role of adequate physical activity and proper diet. The diet of pregnant women should provide the right amount of nutrients and calories. Nutrition standards in Poland are similar to those recommended by World Health Organization (WHO), Institute of Medicine, and European Food Safety Authority (EFSA) [30]. According to Polish guidelines, four to five meals a day are recommended and an energy consumption in the second and third trimesters increased by 360 and 475 kcal/day, respectively. Fats (mainly polyunsaturated fatty acids) should account for 20–35% of the energy requirement, and fat consumption in the second and third trimester should increase by 8–14 and 11–18 g (respectively), compared to the value before pregnancy. Carbohydrate intake should provide 45–60% of calories, but simple sugars should provide no more than 10%. It is recommended that pregnant women consume 1.2 g of protein per kg of body weight (about 54–90 g per day), including animal protein in about 60%. In Poland, 300 g/400 g of fruit/vegetables is the recommended daily amount in the first trimester, and 400 g/500 g in the second–third trimester. The diet should provide the right amount of macro- and micro-nutrients (e.g., vitamins and trace elements) [30]. According to the recommendations of the Institute of Medicine (IOM) of 2009, optimal weight gain depends on the pre-pregnancy BMI, and for underweight, normal weight, overweight, and obesity, it should be 12.5–18, 11.5–16, 7–11.5, and 5–9 kg, respectively [7]. Physical activity is essential in the treatment of obesity. According to WHO, adults should show a minimum of 150 min of moderate activity a week; similar guidelines apply in pregnancy, but with possible contraindications [30].

Our results also showed that gestational weight gain (GWG) was important in predicting GH compared to pre-pregnancy BMI, but GWG may be associated with fluid retention, and this clinical factor had limited predictive value [7,31,32]. In our cohort, the percentage of women with GWG above the IOM recommendation range was 36.8%, but for the women with excessive pre-pregnancy BMI, it was 59.0%.

The mechanisms of the influence of obesity on the higher risk of PE/GH are not fully understood; however, obesity in the mother have been linked to insulin resistance, inflammation (including in the placenta), and restriction of placental blood flow [33]. The increased inflammation and oxidative stress associated with obesity are considered; this may exacerbate the underlying disorders underlying the development of PE [10,34,35,36].

The etiology of pregnancy-induced hypertension is not fully explained, but the pathogenesis of PE takes into account disturbances in trophoblast invasion into the walls of the spiral arteries, which results in a lack of remodeling, high-resistance circulation, and placental ischemia [37,38]. The balance of many placental biomarkers is disturbed, oxidative stress and inflammation in both the placenta and in the mother’s circulation are intensified, and the effect is endothelial function damage and an increase in blood pressure [16,37,38,39]. There are many studies available in the literature which have found associations between lower levels of antioxidants in pregnant women with PE [40]. At the same time, disorders of the woman’s cardiovascular system are taken into account, including factors affecting endothelium (e.g., by increased oxidative stress) [13]. In our previous analyses, we found associations with lower maternal serum selenium (Se) levels (Se being a potent antioxidant) in the first trimester and a higher risk of pregnancy-induced hypertension, including a higher risk of GH [26,34].

In our cohort, the pregnant women had no chronic disease and came from a single region, which matched the study groups in terms of the quality of prenatal care, and ruled out the influence of ethnic/racial differences. The exclusion of high-risk factors (such as multiple pregnancies and inflammatory diseases including preexisting hypertension) resulted in a low number of preeclampsia (PE) cases in our study. We did not separately assess PE phenotypes. Moreover, due to the small number of women with prior PE, we assessed all prior GH/PE cases of hypertension. Adverse neonatal outcomes among our GH and PE cases were significantly more frequent than in normotensive women, but were more severe in the women who developed PE (higher frequency of IUGR cases, lower mean birth weight, and lower mean gestational age, including a much higher percentage of preterm births).

Early identification of women at risk of pregnancy-induced hypertension is needed so they can be placed under increased surveillance as early as possible. Although we did not find such an analysis in the literature, our basic results showing higher GH and/or PE odds ratios for all maternal features tested (Table 1 and Table 2 and Supplementary Materials Tables S2 and S3) are consistent with the results in the literature [41,42,43]. The slightly different hierarchies of maternal factors in GH and PE prediction found in our study may suggest different or common mechanisms of their development.

In the current analysis, “prior GH/PE” was the strongest variable in predicting GH and PE in the assessment of IDI indexes (Table 4 and Table 5) and in the assessment of odds ratios (OR = 22.9 and OR = 27.5, respectively) (Table 2 and Supplementary Materials Table S3). However, when the three predictive indexes (AUC, IDI, and NRI) were summed up, this variable was placed high (but not the highest) in the GH prediction ranking. The weaker final association of this maternal feature with the PE prediction may be due to the very small number of women with prior preeclampsia (PE). The literature results indicate a relationship of prior PE with a higher risk of PE, especially early onset PE (<32th week and <34th week), but not all of these results are based on multivariate analyses [8,9,13,44].

In this analysis, a family history of chronic hypertension was of relatively high importance in predicting PE and GH. When the three measures (AUC, IDI, and NRI) were summed up, family history of hypertension was of greater importance in predicting PE. Importantly, maternal hypertension predicted PE more strongly than paternal hypertension. These results may reflect the influence of genetic factors reported in the literature [9,45,46].

Lower education and lower financial status were other important PE and GH predictors we found. Associations of lower socioeconomic indicators with a higher risk of hypertension in pregnancy are confirmed in the literature [47,48]. Lower socioeconomic indicators may be associated with an unhealthy lifestyle, affecting predictors such as eating unhealthy food, pre-pregnancy obesity, smoking, and lack of adequate prenatal care, including not taking multivitamin supplementation during pregnancy.

In our analysis combining the results of three statistical measures, other risk factors such as longer interpregnancy interval, the lack of multivitamin (and multi-micronutrient) supplementation in the second/third trimester, first trimester smoking, infertility treatment and (separately) in vitro fertilization (IVF), urogenital infection, and hypothyroidism were poorer GH and PE predictors. However, we would like to point out that these factors were associated with higher odds ratios of both forms of the disease and increased the predictive value of the base model (maternal age and primiparity), but less so compared to pre-pregnancy obesity and the factors mentioned above. There are results in the literature confirming the association of these maternal factors with a higher risk of GH and PE [8,47,49,50].

In this analysis, maternal age and primiparity were selected for the primary predictive model and therefore were not assessed separately. Of course, the choice of these two features for the basic predictive model was dictated by the results found in the literature, as both are known risk factors for hypertension in pregnancy [9,20,51,52].

One strength of our study was the prospective cohort study model. An advantage was the exclusion of chronic high-risk factors, which made it possible to assess maternal characteristics as predictors of the disease. Another advantage was being able to confirm the results by using several statistical methods.

The limitation of our study was a small number of preeclampsia cases. We did not assess physical activity or body composition, which would allow additional conclusions to be drawn. The participants self-reported some anthropometric data, but the most important data came from the medical records.

## 5. Conclusions

In this study, the pre-pregnancy BMI was a strong factor in predicting both forms of pregnancy-induced hypertension, i.e., isolated gestational hypertension (GH) and preeclampsia (PE). Pre-pregnancy obesity and overweight were the most likely ones to increase the probability of developing both GH and PE, as compared to other risk factors. Excessive gestational weight gain (GWG) was also an important predictor, which, together with the role of excessive BMI, highlights the urgent need for educational interventions to adhere to guidelines and optimize diet and exercise in women of childbearing age and pregnant women.

In this study, we also found a subtle but distinct difference in the threshold value of BMI in the development of GH and PE, suggesting the need for further research on this topic. It seems important to identify associations of obesity or overweight with various phenotypes of hypertension in pregnancy, which may help in determining the mechanisms of this pathology and in determining the treatment strategy.

This study also showed clear differences in the importance of some risk factors in predicting PE and GH. Hierarchies of PE and GH risk factors may suggest different (or common) mechanisms of their development.

## Figures and Tables

**Table 1:** Basic characteristics of normotensive women and women developing hypertension in pregnancy.

	Controls (*n* = 775)	GH (*n* = 113)		PE (*n* = 24)	
Variables	Mean (SD) or *n* (%)	Mean (SD) or *n* (%)	*p* *	Mean (SD) or *n* (%)	*p* *
Maternal age (years)	33.5 (4.8)	35.0 (4.3)	0.005	34.1 (5.0)	0.434
Primiparous women	318 (41.0%)	53 (46.9%)	0.237	12 (50.0%)	0.380
Prior GH/PE	4 (0.5%)	12 (10.6%)	<0.001	3 (12.5%)	<0.001
Aspirin (for prophylaxis)	7 (0.9%)	2 (1.8%)	0.390	2 (8.3%)	<0.001
Infertility treatment	29 (3.7%)	8 (7.1%)	0.097	3 (12.5%)	0.031
Interpregnancy interval **	4.4 (4.0)	5.6 (4.3)	0.031	7.4 (5.3)	0.023
Smoking in I trimester	37 (4.8%)	17 (15.0%)	<0.001	3 (12.5%)	0.084
Education <12 years **	48 (7%)1	16 (16.3%)	0.002	6 (27.3%)	<0.001
Lower financial status **	95 (26.1%)	28 (43.8%)	<0.001	8 (57.1%)	0.007
Family history					
Hypertension in the mother	137 (17.8%)	31 (27.7%)	0.013	11 (45.8%)	<0.001
Pre-pregnancy BMI (kg/m^2^)	23.3 (4.1)	26.7 (5.3)	<0.001	26.5 (6.2)	0.008
GWG (kg)	13.4 (5.3)	14.6 (8.0)	0.115	15.1(8.2)	0.612
Fetal sex, daughter	370 (47.7%)	58 (51.3%)	0.476	11 (45.8%)	0.853
Gestational age at childbirth (week)	38.9 (1.6)	38.3 (2.2)	0.016	35.1 (3.7)	<0.001
Preterm birth <37th week	41 (5.3%)	11 (9.7%)	0.060	13 (54.2%)	<0.001
Preterm birth <34th week	10 (1.3%)	7 (6.2%)	<0.001	6 (25.0%)	<0.001
Newborn birthweight (g)	3416.5 (511.7)	3174.1 (734.3)	0.001	2294.2 (927.5)	<0.001
IUGR cases	11 (1.4%)	6 (5.5%)	0.004	4 (18.2%)	<0.001
Cesarean section	306 (39.5%)	54 (47.8%)	0.093	22 (91.7%)	<0.001
GDM	121 (15.6%)	22 (19.5%)	0.298	3 (12.5%)	0.678
Gestational diabetes mellitus	121 (15.6%)	22 (19.5%)	0.298	3 (12.5%)	0.678
PE beginning <32th week				7 (29.2%)	
PE beginning ≥34th week	-	-	-	13 (54.2%)	-
Blood pressure before pregnancy					
Systolic (mmHg)	104.9 (10.4)	123.5 (8.5)	<0.001	123.3 (8.7)	<0.001
Diastolic (mmHg)	64.5 (8.7)	76.8 (7.7)	<0.001	78.1 (8.6)	<0.001
Blood pressure after delivery					
Systolic (mmHg)	107.9 (10.8)	157.2 (17.6)	<0.001	170.3 (17.5)	<0.001
Diastolic (mmHg)	66.8 (8.8)	99.6 (9.3)	<0.001	106.8 (15.8)	<0.001

* The Mann–Whitney U test was used for comparisons of continuous variables, and the Pearson chi-square test (or Fisher exact test when Cochran assumption was not met) for binomial categories was used (*p* < 0.05 was assumed to be significant); ** for available data. Controls = normotensive women; GH, gestational hypertension; PE, preeclampsia; BMI, body mass index; GWG, gestational weight gain; IUGR, Intrauterine Growth Restriction; GDM, gestational diabetes mellitus.

**Table 2:** The odds ratios of gestational hypertension (GH) and preeclampsia (PE) for selected maternal characteristics.

Variables	GH OR (95% CI) *	*p* **	PE OR (95% CI) *	*p* **
Continuous variables:				
Pre-pregnancy BMI (kg/m^2^)	1.16 (1.11–1.21)	<0.001	1.15 (1.06–1.24)	<0.001
Maternal age (years)	1.07 (1.03–1.12)	0.003	1.03 (0.94–1.12)	0.539
GWG (kg)	1.04 (1.00–1.07)	0.040	1.06 (0.98–1.14)	0.145
Other variables:				
Prior GH/PE (vs. others)	22.90 (7.3–72.4)	<0.001	27.54 (5.8–130.8)	<0.001
BMI ≥ 30 kg/m^2^ (vs. normal BMI)	5.60 (3.32–9.43)	<0.001	9.21 (3.52–24.11)	<0.001
Smoking in I trimester (vs. others)	3.53 (1.92–6.52)	<0.001	2.85 (0.81–9.99)	0.102
Age ≥40 years (vs. 25–29 years)	3.23 (1.41–7.38)	0.005	1.11 (0.1–12.49)	0.933
Education <12 years vs. others **	2.50 (1.37–4.57)	0.003	5.05 (1.92–13.31)	0.001
GWG above the range (vs. normal)	2.45 (1.53–3.92)	<0.001	1.57 (0.62–3.97)	0.337
Financial status (1-2-3) vs. others **	2.36 (1.46–3.80)	<0.001	3.58 (1.49–8.59)	0.004
Urogenital infection (vs. others)	2.12 (1.31–3.43)	0.002	0.61 (0.14–2.65)	0.513
No multivitamins (vs. others) #	2.11 (1.41–3.15)	<0.001	0.90 (0.39–2.08)	0.806
Family history of Hypertension (H)				
H in the father (vs. controls) ***	2.06 (1.29–3.28)	0.003	1.84 (0.62–5.47)	0.274
H in the mother (vs. controls) ***	1.90 (1.18–3.06)	0.008	3.98 (1.66–9.57)	0.002
Interpregnancy interval (years)				
≥11 years (vs. 1 year)	2.02 (0.90–4.58)	0.091	6.63 (1.18–37.29)	0.032
Hypothyroidism (vs. others)	2.01 (1.23–3.29)	0.006	2.36 (0.91–6.09)	0.076
Infertility treatment (vs. others)	1.96 (0.87–4.4)	0.103	3.68 (1.04–13.03)	0.044
In vitro fertilization (vs. others)	1.92 (0.76–4.84)	0.167	3.11 (0.69–14.06)	0.140
Primiparity (vs. multiparity)	1.27 (0.85–1.89)	0.238	1.22 (0.54–2.75)	0.638

* OR, crude odds ratios (and 95% confidence intervals) calculated in unidimensional logistic regression; ** *p*-value < 0.05 was statistically significant; *** controls = women without family history of hypertension. # No multivitamins supplementation in 2nd-3rd trimester. GH, gestational hypertension; PE, preeclampsia; BMI, body mass index; GWG, gestational weight gain; H, hypertension.

**Table 3:** Area under curve (AUC) values for predicting GH and PE.

Variables	GH AUC	*p* *	PE AUC	*p* *
Pre-pregnancy BMI (kg/m^2^)	0.698	<0.001	0.660	0.008
Maternal age (years)	0.581	0.005	0.547	0.435
GWG (kg)	0.546	0.115	0.530	0.613
Maternal age + primiparity	0.600	0.001	0.539	0.520

* A *p*-value < 0.05 was statistically significant. GH, gestational hypertension; PE, preeclampsia; AUC, area under receiver operating characteristic curve; BMI, body mass index; GWG, gestational weight gain.

**Table 4:** Values of the three predictive indicators in the extended multivariate models in the assessment of the probability of gestational hypertension (GH).

			GH			
Base Model (Maternal Age + Primiparity)	AUC Base 0.600					
Extended Models (Base Model + Listed Variables)	AUC Extended	*p*-Value * (Extended vs. Base)	IDI	*p*-Value * (Extended vs. Base)	NRI	*p*-Value * (Extended vs. Base)
Pre-pregnancy BMI (kg/m^2^)	0.716	<0.001	0.064	<0.001	0.542	<0.001
Pre-pregnancy BMI (c)	0.704	<0.001	0.058	<0.001	0.571	<0.001
Pre-pregnancy weight (kg)	0.697	<0.001	0.044	<0.001	0.544	<0.001
Pre-pregnancy BMI ≥ 25 kg/m^2^	0.685	0.001	0.041	<0.001	0.571	<0.001
Pre-pregnancy BMI ≥ 30 kg/m^2^	0.663	0.002	0.045	<0.001	0.399	<0.001
Prior GH/PE	0.656	0.002	0.068	<0.001	0.433	<0.001
GWG (c)	0.653	0.024	0.027	<0.001	0.419	<0.001
Financial status	0.648	0.022	0.015	0.007	0.250	0.003
No multivitamins	0.646	0.039	0.016	0.001	0.368	<0.001
Smoking in I trimester	0.646	0.025	0.024	0.003	0.205	0.003
Education <12 years	0.643	0.018	0.01	0.043	0.159	0.019
Family history; H in the mother (c)	0.639	0.034	0.004	0.19	0.236	0.019
Family history; H in the father (c)	0.638	0.052	0.008	0.046	0.279	0.005
No folic acid supplementation	0.634	0.101	0.013	<0.001	0.312	<0.001
Urogenital infection	0.632	0.075	0.01	0.022	0.220	0.009
Family history of Hypertension	0.631	0.064	0.004	0.194	0.236	0.019
Interpregnancy interval (c)	0.619	0.166	0.004	0.086	−0.014	0.891
Hypothyroidism	0.618	0.253	0.008	0.032	0.195	0.017
GWG (kg)	0.616	0.402	0.011	0.009	0.149	0.139
Maternal height (cm)	0.613	0.22	0.003	0.137	0.125	0.213
Place of residence (c)	0.611	0.318	0.002	0.238	0.144	0.094
GWG > 15 kg	0.607	0.551	0.004	0.077	0.113	0.248
Treatment of infertility	0.601	0.621	0.0007	0.562	−0.074	0.386
In vitro fertilization	0.6	0.925	0.0005	0.6	−0.12	0.151
GWG > 10 kg	0.598	0.599	0.0003	0.454	−0.011	0.903

* A *p*-value < 0.05 was statistically significant. AUC, area under receiver operating characteristic curve; IDI, Integrated Discrimination Improvement; NRI, Net Reclassification Improvement; (c): categories of independent variables (described in the Methodology); BMI, body mass index; GWG, gestational weight gain; H, hypertension in family history.

**Table 5:** Values of the three predictive indicators in the extended multivariate models in the assessment of the probability of preeclampsia (PE).

			PE			
Base Model (Maternal Age + Primiparity)	AUC Base 0.539					
Extended Models (Base Model + Listed Variables)	AUC Extended	*p*-Value *(Extended vs. Base)	IDI	*p*-Value * (Extended vs. Base)	NRI	*p*-Value * (Extended vs. Base)
Pre-pregnancy BMI (c)	0.726	0.017	0.034	0.002	0.688	0.001
Family history of H in the mother (c)	0.717	0.009	0.013	0.020	0.563	0.006
Pre-pregnancy BMI ≥ 30 kg/m^2^	0.703	0.009	0.032	0.004	0.600	0.002
Pre-pregnancy BMI ≥ 25 kg/m^2^	0.679	0.042	0.012	0.011	0.575	0.005
Pre-pregnancy BMI (kg/m^2^)	0.678	0.107	0.02	0.019	0.510	0.013
Family history of H in the father (c)	0.658	0.094	0.007	0.059	0.447	0.029
Financial status	0.648	0.068	0.012	0.039	0.422	0.030
Interpregnancy interval (c)	0.646	0.138	0.007	0.072	0.405	0.032
Pre-pregnancy weight (kg)	0.642	0.233	0.012	0.041	0.373	0.071
Family history of hypertension (H)	0.638	0.119	0.005	0.069	0.447	0.029
Education <12 years	0.629	0.05	0.018	0.034	0.376	0.034
No folic acid supplementation	0.625	0.032	0.005	0.010	0.373	0.028
Prior GH/PE	0.622	0.097	0.050	0.080	0.098	0.602
Maternal height (cm)	0.589	0.289	0.002	0.253	0.157	0.448
Smoking in I trimester	0.588	0.319	0.004	0.266	0.155	0.255
GWG (c)	0.586	0.407	0.002	0.205	0.238	0.249
Place of residence (c)	0.586	0.187	0.001	0.161	0.174	0.302
Treatment of infertility	0.585	0.163	0.005	0.251	−0.121	0.436
Urogenital infection	0.568	0.188	0.0008	0.258	0.091	0.428
GWG (kg)	0.565	0.72	0.006	0.165	0.055	0.791
GWG > 15 kg	0.563	0.507	0.0004	0.577	0.084	0.675
No multivitamins supplementation	0.557	0.319	−0.0001	0.870	0.05	0.803
In vitro fertilization	0.555	0.174	0.003	0.372	−0.183	0.239
GWG > 10 kg	0.555	0.49	0.0001	0.792	0.094	0.631
Hypothyroidism	0.553	0.655	0.005	0.124	0.252	0.157

* A *p*-value < 0.05 was statistically significant. AUC, area under receiver operating characteristic curve; IDI, Integrated Discrimination Improvement; NRI, Net Reclassification Improvement; (c), categories of independent variables (described in the Methodology); BMI, body mass index; H, hypertension in family history; GWG, gestational weight gain.

## Supplementary Materials

The following are available online at https://www.mdpi.com/2072-6643/12/9/2681/s1. Figure S1: IDI and NRI index values after extending the predictive base model with the abovementioned maternal characteristics. Table S1: Basic characteristics of the women with excessive pre-pregnancy BMI. Table S2: Complete characteristics of normotensive women and women developing hypertension in pregnancy. Table S3: Set of odds ratios of pregnancy hypertension forms for many categories of the mother’s features. Table S4: Set of AUC values in the extended multivariate models in the assessment of the probability of gestational hypertension (GH) and preeclampsia (PE). Table S5: Set of values of Net Reclassification Improvement (NRI) in the extended multivariate models in the assessment of the probability of gestational hypertension (GH) and preeclampsia (PE). Table S6: Set of values of Integrated Discrimination Improvement (IDI) in the extended multivariate models in the assessment of the probability of gestational hypertension (GH) and preeclampsia (PE).
