# Supplementary material for: Pre-Pregnancy Obesity vs. Other Risk Factors in Probability Models of Preeclampsia and Gestational Hypertension
Source: Nutrients. 2020 Sep 2;12(9):2681. doi: 10.3390/nu12092681 (PMC7551880; doi:10.3390/nu12092681)
Supplement: Supplementary file 1 [file nutrients-12-02681-s001.zip › Table S1.docx]

**Table S1.** Basic characteristics of the women with excessive pre-pregnancy BMI

|  | **Median (IQR) or n (%)** | **Median (IQR) or n (%)** | **p*** |
| --- | --- | --- | --- |
| **Characteristics** | **Normal**  **Pre-pregnancy BMI**  **(n = 593)** | **BMI ≥ 25 kg / m²**  **(n = 271)** |  |
| Overweight | - | 173 |  |
| Obesity ≥ 30 kg / m² | - | 98 |  |
| Obesity I degree (BMI 30.0-34.9) | - | 70 |  |
| Obesity II degree (BMI 35-39.9) | - | 25 |  |
| Obesity III degree (BMI ≥ 40 kg/m²) | - | 4 |  |
| Pre-pregnancy weight (kg) | 60 (55-65) | 81 (75-89) | <0.0001 |
| Pre-pregnancy BMI (kg/m²) | 21.7 (20.3-23.2) | 28.7 (26.7-31.3) | <0.0001 |
| GWG (kg) | 14 (11-17) | 12 (8-17) | 0.0014 |
| Categories |  |  | <0.0001 |
| GWG above the range | 170 (28.7%) | 160 (59%) |  |
| GWG in the range | 246 (41.5%) | 72 (26.6%) |  |
| GWG below the range | 177 (29.8%) | 39 (14.4%) |  |
| Primiparous women | 250 (42.2%) | 101 (37.3%) | 0.1746 |
| Maternal age | 35 (30-37) | 35 (32-37) | 0.0029 |
| PIH | 60 (10.1%) | 74 (27.3%) | <0.0001 |
| GDM | 79 (13.3%) | 58 (21.4%) | 0.003 |
|  | **Normal**  **Pre-pregnancy BMI**  **(n = 593)** | **Obesity**  **(BMI ≥ 30 kg/m²)**  **(n = 98)** |  |
| Pre-pregnancy weight (kg) | 60 (55-65) | 90 (87-97) | <0.0001 |
| Pre-pregnancy BMI (kg/m²) | 21.7 (20.3-23.2) | 32.7 (31.1-35.3) | <0.0001 |
| GWG (kg) | 14 (11-17) | 11 (7-16) | <0.0001 |
| Categories |  |  | <0.0001 |
| GWG above the range | 170 (28.7%) | 54 (55.1%) |  |
| GWG in the range | 246 (41.5%) | 25 (25.5%) |  |
| GWG below the range | 177 (29.8%) | 19 (19.4%) |  |
| Primiparous women | 250 (42.2%) | 39 (39.8%) | 0.661 |
| Maternal age | 35 (30-37) | 36 (33-38) | 0.004 |
| PIH | 60 (10.1%) | 40 (40.8%) | <0.0001 |
| GDM | 79 (13.3%) | 32 (32.7%) | <0.0001 |

* The Mann-Whitney U test was used for comparisons of continuous variables, For categorical ordered categories Cochran-Armitage test for trend was calculated, and for binomial categories the Pearson chi-square test (or Fisher exact test when Cochran assumption was not met) was used (p<0.05 was assumed to be significant). BMI: body mass index; GWG: gestational weight gain; PIH: pregnancy-induced hypertension GDM: gestational diabetes mellitus.
