# Supplementary material for: Pre-Pregnancy Obesity vs. Other Risk Factors in Probability Models of Preeclampsia and Gestational Hypertension
Source: Nutrients. 2020 Sep 2;12(9):2681. doi: 10.3390/nu12092681 (PMC7551880; doi:10.3390/nu12092681)
Supplement: Supplementary file 1 [file nutrients-12-02681-s001.zip › Table S2.docx]

**Table S2.** Complete characteristics of normotensive women and women developing hypertension in pregnancy.

|  | **Controls**  **(n = 775)** | **GH**  **(n = 113)** |  | **PE**  **(n = 24)** |  |
| --- | --- | --- | --- | --- | --- |
| **Variables** | **Mean (SD)**  **or n (%)** | **Mean (SD)**  **or n (%)** | **p *** | **Mean (SD)**  **or n (%)** | **p *** |
| Maternal age (years) | 33.5 (4.8) | 35.0 (4.3) |  | 34.1 (5.0) |  |
| Median | 35.0 | 36.0 | 0.005 | 35.0 | 0.434 |
| < 20 | 4 (0.5%) | 1 (0.9%) | 0.625 | 0 (0%) | - |
| 20-24 | 32 (4.1%) | 1 (0.9%) | 0.089 | 2 (8.3%) | 0.315 |
| 25-29 | 131 (16.9%) | 11 (9.7%) | 0.052 | 2 (8.3%) | 0.267 |
| 30-34 | 216 (27.9%) | 28 (24.8%) | 0.491 | 5 (20.8%) | 0.448 |
| 35-39 | 333 (43.0%) | 56 (49.6%) | 0.187 | 14 (58.3%) | 0.135 |
| 40 | 59 (7.6%) | 16 (14.2%) | 0.019 | 1 (4.2%) | 0.528 |
| Primiparous women | 318 (41.0%) | 53 (46.9%) | 0.237 | 12 (50.0%) | 0.380 |
| Aspirin (for prophylaxis) | 7 (0.9%) | 2 (1.8%) | 0.390 | 2 (8.3%) | < 0.001 |
| Prior GH/PE | 4 (0.5%) | 12 (10.6%) | < 0.001 | 3 (12.5%) | < 0.001 |
| Infertility treatment | 29 (3.7%) | 8 (7.1%) | 0.097 | 3 (12.5%) | 0.031 |
| In vitro fertilization | 22 (2.8%) | 6 (5.3%) | 0.160 | 2 (8.3%) | 0.120 |
| Interpregnancy interval (years) ** | 4.4 (4.0) | 5.6 (4.3) |  | 7.4 (5.3) |  |
| Median | 3.0 | 5.0 | 0.031 | 7.5 | 0.023 |
| Missing data, n = 310  Primigravida, n = 280 |  |  |  |  |  |
| Multivitamins in II-III trimester | 465 (60%) | 47 (41.6%) | <0.001 | 15 (62.5%) | 0.806 |
| Urogenital infection | 100 (12.9%) | 27 (23.9%) | 0.002 | 2 (8.3%) | 0.509 |
| Hypothyroidism | 96 (12.4%) | 25 (22.1%) | 0.005 | 6 (25%) | 0.068 |
| Smoking in I trimester | 37 (4.8%) | 17 (15.0%) | < 0.001 | 3 (12.5%) | 0.084 |
| Pre-pregnancy BMI (kg/m²) | 23.3 (4.1) | 26.7 (5.3) |  | 26.5 (6.2) |  |
| Median | 22.5 | 25.5 | < 0.001 | 25.0 | 0.008 |
| Underweight | 44 (5.7%) | 1 (0.9%) | 0.030 | 2 (8.3%) | 0.582 |
| Normal BMI | 533 (68.8%) | 51 (45.1%) | < 0.001 | 9 (37.5%) | 0.001 |
| Overweight | 139 (17.4%) | 30 (26.6%) | 0.029 | 4 (16.7%) | 0.873 |
| Obesity | 58 (7.5%) | 31 (27.4%) | < 0.001 | 9 (37.5%) | < 0.001 |
| GWG (kg) | 13.4 (5.3) | 14.6 (8.0) |  | 15.1 (8.2) |  |
| Median | 13.0 | 14.0 | 0.115 | 14.3 | 0.612 |
| GWG below the range | 211 (27.2%) | 22 (19.5%) | 0.080 | 5 (20.8%) | 0.487 |
| GWG in the range | 301 (38.8%) | 29 (25.7%) | 0.007 | 8 (33.3%) | 0.586 |
| GWG over the range | 263 (33.9%) | 62 (54.9%) | < 0.001 | 11 (45.8%) | 0.227 |
| Family history of hypertension |  |  |  |  |  |
| Hypertension in the father | 135 (17.5%) | 33 (29.5%) | 0.003 | 5 (20.8%) | 0.673 |
| Hypertension in the mother | 137 (17.8%) | 31 (27.7%) | 0.013 | 11 (45.8%) | < 0.001 |
| Education < 12 years ** | 48 (7.%)1 | 16 (16.3%) | 0.002 | 6 (27.3%) | < 0.001 |
| Missing data, n = 114 |  |  |  |  |  |
| Place of residence, village | 234 (30.3%) | 26 (23%) | 0.113 | 8 (33.3%) | 0.748 |
| Place of residence, big city | 312 (40.4%) | 50 (44.3%) | 0.433 | 11 (45.8%) | 0.591 |
| Lower financial status 1-2-3 ** | 95 (26.1%) | 28 (43.8%) | <0.001 | 8 (57.1%) | 0.007 |
| Missing data, n = 422 |  |  |  |  |  |
| Gestational age at delivery (week) | 38.9 (1.6) | 38.3 (2.2) |  | 35.1 (3.7) |  |
| Median | 39.0 | 39.0 | 0.016 | 36.0 | < 0.001 |
| Newborn birthweight (g) | 3416.5 (511.7) | 3174.1 (734.3) |  | 2294.2 (927.5) |  |
| Median | 3449.0 | 3200.0 | 0.001 | 2445.0 | < 0.001 |
| Gestational diabetes mellitus (GDM) | 121 (15.6%) | 22 (19.5%) | 0.298 | 3 (12.5%) | 0.678 |
| PE beginning < 32th week | - | - | - | 7 (29.2%) | - |
| PE beginning ≥ 34th week | - | - | - | 13 (54.2%) | - |

* The Mann-Whitney U test was used for comparisons of continuous variables, and the Pearson chi-square test (or Fisher exact test when Cochran assumption was not met) for binomial categories was used (p<0.05 was assumed to be significant); ** For available data. Controls: normotensive women; GH: gestational hypertension; PE: preeclampsia; BMI: body mass index; GWG: gestational weight gain.
