# Supplementary material for: Pre-Pregnancy Obesity vs. Other Risk Factors in Probability Models of Preeclampsia and Gestational Hypertension
Source: Nutrients. 2020 Sep 2;12(9):2681. doi: 10.3390/nu12092681 (PMC7551880; doi:10.3390/nu12092681)
Supplement: Supplementary file 1 [file nutrients-12-02681-s001.zip › Table S3.docx]

**Table S3.** Set of odds ratios of pregnancy hypertension forms for many categories of the mother's features.

|  | **GH** |  | **PE** |  | | | |  |
| --- | --- | --- | --- | --- | --- | --- | --- | --- |
| **Variables / risk factors** | **OR (95% CI) *** | **p **** | **OR (95% CI) *** | | **p **** | | |  |
| Pre-pregnancy BMI (kg/m²) | 1.16 (1.11-1.21); | < 0.001 | 1.15 (1.06-1.24); | | < 0.001 | | |  |
| Underweight (vs Ref) | 0.24 (0.03-1.76); | 0.160 | 2.70 (0.57-12.87); | | 0.213 | | |  |
| Normal BMI | Ref |  | Ref | |  | | |  |
| Overweight (vs Ref) | 2.26 (1.39-3.68); | 0.001 | 1.71 (0.52-5.63); | | 0.379 | | |  |
| Obesity (vs Ref) | 5.60 (3.32-9.43); | < 0.001 | 9.21 (3.52-24.11); | | < 0.001 | | |  |
| Pre-pregnancy weight (kg) | 1.04 (1.03-1.06); | < 0.001 | 1.04 (1.01-1.07); | | 0.005 | | |  |
| Maternal height (cm) | 0.98 (0.95-1.01); | 0.203 | 0.96 (0.9-1.03); | | 0.252 | | |  |
| Prior GH/PE (vs others) | 22.90 (7.25-72.36); | < 0.001 | 27.54 (5.8-130.83); | | < 0.001 | | |  |
| Maternal age (years) | 1.07 (1.03-1.12); | 0.003 | 1.03 (0.94-1.12); | | 0.539 | | |  |
| [18-24] vs Ref | 0.66 (0.14-3.12); | 0.602 | 3.64 (0.5-26.74); | | 0.204 | | |  |
| [25-29] | Ref |  | Ref | |  | | |  |
| [30-34] vs Ref | 1.54 (0.74-3.21); | 0.244 | 1.52 (0.29-7.93); | | 0.622 | | |  |
| [35-39] vs Ref | 2.00 (1.02-3.94); | 0.044 | 2.75 (0.62-12.28); | | 0.184 | | |  |
| [≥ 40] vs Ref | 3.23 (1.41-7.38); | 0.005 | 1.11 (0.1-12.49); | | 0.933 | | |  |
| Primiparity vs multiparity | 1.27 (0.85-1.89); | 0.238 | 1.22 (0.54-2.75); | | 0.638 | | |  |
| Interpregnancy interval [c] [1 year] | Ref |  | Ref | |  | | |  |
| Interpregnancy int. [c][2] vs Ref | 0.79 (0.3-2.08); | 0.635 | 1.19 (0.11-13.31); | | 0.890 | | |  |
| Interpregnancy int. [c][3-5] vs Ref | 1.22 (0.6-2.47); | 0.578 | 1.94 (0.32-11.79); | | 0.472 | | |  |
| Interpregnancy int. [c][6-10] vs Ref | 1.65 (0.84-3.27); | 0.150 | 2.97 (0.54-16.51); | | 0.213 | | |  |
| Interpregnancy int. [c][≥11] vs Ref | 2.02 (0.9-4.58); | 0.091 | 6.63 (1.18-37.29); | | 0.032 | | |  |
| Infertility treatment (vs others) | 1.96 (0.87-4.4); | 0.103 | 3.68 (1.04-13.03); | | 0.044 | | |  |
| In vitro fertilization (vs others) | 1.92 (0.76-4.84); | 0.167 | 3.11 (0.69-14.06); | | 0.140 | | |  |
| Urogenital infection (vs others) | 2.12 (1.31-3.43); | 0.002 | 0.61 (0.14-2.65); | | 0.513 | | |  |
| Hypothyroidism (vs others) | 2.01 (1.23-3.29); | 0.006 | 2.36 (0.91-6.09); | | 0.076 | | |  |
| Smoking in I trimester (vs others) | 3.53 (1.92-6.52); | < 0.001 | 2.85 (0.81-9.99); | | 0.102 | | |  |
| No folic acid supplementation in I trimester (vs others) | 2.08 (1.32-3.28); | 0.002 | 2.48 (0.92-6.71); | | 0.074 | | |  |
| No multivitamins in II-III trimester  (vs others) | 2.11 (1.41-3.15); | < 0.001 | 0.90 (0.39-2.08); | | 0.806 | | |  |
| GWG (kg) | 1.04 (1-1.07); | 0.040 | 1.06 (0.98-1.14); | | 0.145 | | |  |
| GWG >10 kg (vs ≤ 10 kg) | 0.97 (0.63-1.5); | 0.902 | 0.80 (0.34-1.9); | | 0.618 | | |  |
| GWG >15 kg (vs ≤15 kg) | 1.28 (0.85-1.92); | 0.237 | 1.20 (0.52-2.78); | | 0.667 | | |  |
| GWG below the range vs Ref | 1.08 (0.61-1.94); | 0.790 | 0.89 (0.29-2.76); | | 0.842 | | |  |
| GWG in the range | Ref |  | Ref | |  | | |  |
| GWG above the range vs Ref | 2.45 (1.53-3.92); | < 0.001 | 1.57 (0.62-3.97); | | 0.337 | | |  |
| Family history of hypertension |  |  |  | |  | | |  |
| No hypertension in the family | Ref |  | Ref | |  | | |  |
| Hypertension in the mother (vs Ref) | 1.90 (1.18-3.06); | 0.008 | 3.98 (1.66-9.57); | | 0.002 | | |  |
| Hypertension in the father (vs Ref) | 2.06 (1.29-3.28); | 0.003 | 1.84 (0.62-5.47); | | 0.274 | | |  |
| Education. <12 years (vs ≥ 12 years) | 2.50 (1.37-4.57); | 0.003 | 5.05 (1.92-13.31); | | 0.001 | | |  |
| Big city (vs Village) | 1.44 (0.87-2.39); | 0.154 | 1.03 (0.41-2.6); | | 0.948 | | |  |
| Financial status [1-2-3] (vs. 4-5) | 2.36 (1.46-3.8); | < 0.001 | 3.58 (1.49-8.59); | | 0.004 | | |  |
| Blood pressure before pregnancy |  |  |  | |  | | |  |
| Systolic (mmHg) | 1.20 (1.17-1.24); | < 0.001 | 1.18 (1.12-1.24); | | < 0.001 | | |  |
| Diastolic (mmHg) | 1.17 (1.14-1.2); | < 0.001 | 1.18 (1.12-1.25); | | < 0.001 | | |  |
| Fetal sex, daughter (vs son) | 1.15(0.78-1.71) | 0.476 | 0.93(0.41-2.09) | | 0.854 | | |  |
| GDM (vs no GDM) | 1.31(0.79-2.16) | 0.299 | 0.77(0.23-2.63) | | 0.679 | | |  |
| GDM-2 (vs no GDM) | 1.35(0.39-4.71) | 0.641 | 3.89(0.84-18.03) | | 0.082 | | |  |
| Maternal height < 164 cm (vs 164-171) | 1.29(0.82-2.03) | 0.269 | 1.41(0.56-3.56) | | 0.468 | | |  |
| Maternal height > 171 cm (vs 164-171) | 1.05(0.62-1.76) | 0.864 | 1.12(0.38-3.26) | | 0.840 | | |  |
| **Variables / risk factors** | **AOR-a (95% CI) *** | **p **** | **AOR-a (95% CI) *** | | | **p **** | | |
| Continuous variables: |  |  |  | | | |  | |
| Pre-pregnancy BMI (kg/m²) | 1.16(1.11-1.20) | < 0.001 | 1.15(1.06-1.24) | | | | <0.001 | |
| Maternal age (years) | 1.09(1.04-1.14) | <0.001 | 1.04(0.95-1.15) | | | | 0.400 | |
| GWG (kg) | 1.05(1.01-1.08) | 0.013 | 1.06(0.99-1.14) | | | | 0.113 | |
| Other variables: |  |  |  | | | |  | |
| Prior GH/PE (vs others) | 30.04(9.19-98.12) | <0.001 | 32.77(6.44-166.84) | | | | <0.001 | |
| BMI ≥ 30 kg/m² (vs normal BMI) | 5.20(3.06-8.84) | < 0.001 | 9.01(3.42-23.73) | | | | <0.001 | |
| Smoking in I trimester (vs others) | 4.26(2.25-8.04) | < 0.001 | 3.18(0.89-11.31) | | | | 0.075 | |
| Age ≥ 40 years (vs 25-29 years) | 3.23(1.41-7.38) | 0.006 | 1.32(0.11-15.49) | | | | 0.827 | |
| Education < 12 years vs. others ** | 2.76(1.48-5.15) | 0.001 | 5.44(2.01-14.71) | | | | 0.001 | |
| GWG above the range (vs normal) | 2.55(1.58-4.10) | < 0.001 | 1.62(0.64-4.1) | | | | 0.308 | |
| Financial status (1-2-3) vs others ** | 2.53(1.56-4.12) | < 0.001 | 3.64(1.51-8.76) | | | | 0.004 | |
| Urogenital infection (vs others) | 2.08(1.27-3.38) | 0.003 | 0.60(0.14-2.58) | | | | 0.489 | |
| No multivitamins (vs others) # | 2.10(1.4-3.16) | < 0.001 | 0.90(0.39-2.09) | | | | 0.804 | |
| Family history of Hypertension (H) |  |  |  | | | |  | |
| H in the father (vs controls) *** | 2.02(1.26-3.24) | 0.004 | 1.78(0.60-5.31) | | | | 0.301 | |
| H in the mother (vs controls) *** | 1.79(1.11-2.88) | 0.018 | 3.90(1.60-9.47) | | | | 0.003 | |
| Interpregnancy interval (years) |  |  |  | | | |  | |
| ≥ 11 years (vs 1 year) | 1.73(0.73-4.14) | 0.216 | 6.69(1.08-41.61) | | | | 0.041 | |
| Hypothyroidism (vs others) | 1.88(1.14-3.10) | 0.014 | 2.27(0.88-5.91) | | | | 0.092 | |
| Infertility treatment (vs others) | 1.34(0.57-3.13) | 0.499 | 3.28(0.84-12.77) | | | | 0.087 | |
| In vitro fertilization (vs others) | 1.28(0.49-3.36) | 0.611 | 2.64(0.54-12.97) | | | | 0.231 | |
| Primiparity (vs multiparity) | 1.65(1.08-2.51) | 0.020 | 1.40(0.58-3.35) | | | | 0.452 | |
| Age ≥ 35 years vs others | 1.93(1.26-2.96) | 0.003 | 1.82(0.76-4.39) | | | | 0.180 | |
| Fetal sex, daughter (vs son) | 1.13(0.76-1.68) | 0.553 | 0.91(0.4-2.07) | | | | 0.828 | |
| GDM (vs no GDM) | 1.14(0.68-1.9) | 0.626 | 0.7(0.2-2.41) | | | | 0.566 | |
| GDM-2 (vs no GDM) | 1.3(0.37-4.61) | 0.68 | 3.88(0.82-18.25) | | | | 0.086 | |
| Maternal height < 164 cm (vs 164-171) | 1.35(0.86-2.14) | 0.195 | 1.46(0.58-3.69) | | | | 0.427 | |
| Maternal height > 171 cm (vs 164-171) | 1.05(0.62-1.77) | 0.86 | 1.12(0.38-3.27) | | | | 0.838 | |
| **Variables / risk factors** | **AOR-b (95% CI) *** | **p **** | **AOR-b (95% CI) *** | | | **p **** | | |
| Continuous variables: |  |  |  | | | |  | |
| Pre-pregnancy BMI (kg/m²) | 1.16(1.11-1.20) | <0.001 | 1.15(1.07-1.25) | | | | <0.001 | |
| Maternal age (years) | 1.62(1.04-2.51) | 0.032 | 1.02(0.93-1.12) | | | | 0.680 | |
| GWG (kg) | 1.06(1.03-1.10) | 0.001 | 1.07(1.00-1.14) | | | | 0.051 | |
| Other variables: |  |  |  | | | |  | |
| Prior GH/PE (vs others) | 34.06(10.12-114.63) | <0.001 | 32.77(6.11-175.62) | | | | <0.001 | |
| BMI ≥ 30 kg/m² (vs normal BMI) | 5.20(3.06-8.84) | <0.001 | 9.01(3.42-23.73) | | | | <0.001 | |
| Smoking in I trimester (vs others) | 3.67(1.87-7.18) | <0.001 | 2.81(0.77-10.18) | | | | 0.117 | |
| Age ≥ 40 years (vs 25-29 years) | 3.01(1.24-7.33) | 0.015 | 1.01(0.08-12.28) | | | | 0.992 | |
| Education < 12 years vs. others ** | 1.91(0.98-3.75) | 0.059 | 4.37(1.54-12.45) | | | | 0.006 | |
| GWG above the range (vs normal) | 1.97(1.02-3.23) | 0.007 | 1.24(0.48-3.20) | | | | 0.653 | |
| Financial status (1-2-3) vs others ** | 1.70(1.01-2.87) | 0.047 | 2.54(1.01-6.43) | | | | 0.048 | |
| Urogenital infection (vs others) | 2.05(1.23-3.41) | 0.006 | 0.53(0.12-2.35) | | | | 0.406 | |
| No multivitamins (vs others) # | 2.27(1.49-3.47) | <0.001 | 0.93(0.40-2.18) | | | | 0.871 | |
| Family history of Hypertension (H) |  |  |  | | | |  | |
| H in the father (vs controls) *** | 2.02(1.24-3.29) | 0.005 | 1.68(0.56-5.04) | | | | 0.357 | |
| H in the mother (vs controls) *** | 1.53(0.93-2.53) | 0.094 | 3.29(1.34-8.12) | | | | 0.010 | |
| Interpregnancy interval (years) |  |  |  | | | |  | |
| ≥ 11 years (vs 1 year) | 1.44(0.58-3.53) | 0.431 | 5.05(0.8-31.98) | | | | 0.085 | |
| Hypothyroidism (vs others) | 1.49(0.88-2.52) | 0.140 | 1.78(0.67-4.73) | | | | 0.250 | |
| Infertility treatment (vs others) | 1.50(0.62-3.64) | 0.365 | 3.98(0.99-15.96) | | | | 0.051 | |
| In vitro fertilization (vs others) | 1.50(0.55-4.08) | 0.431 | 3.27(0.63-16.89) | | | | 0.157 | |
| Primiparity (vs multiparity) | 1.76(1.14-2.73) | 0.011 | 1.32(0.57-3.03) | | | | 0.517 | |
| Age ≥ 35 years vs others | 1.62(1.04-2.51) | 0.032 | 1.51(0.62-3.65) | | | | 0.366 | |
| Fetal sex, daughter (vs son) | 1.13(0.75-1.70) | 0.575 | 0.96(0.42-2.18) | | | | 0.913 | |
| GDM (vs no GDM) | 0.85(0.49-1.46) | 0.553 | 0.48(0.13-1.74) | | | | 0.264 | |
| GDM-2 (vs no GDM) | 0.72(0.19-2.81) | 0.641 | 2.1(0.39-11.32) | | | | 0.387 | |
| Maternal height < 164 cm (vs 164-171) | 1.36(0.84-2.18) | 0.210 | 1.54(0.60-3.96) | | | | 0.368 | |
| Maternal height > 171 cm (vs 164-171) | 1.05(0.61-1.81) | 0.854 | 1.15(0.39-3.41) | | | | 0.796 | |

* crude (OR) odds ratios (and 95% confidence intervals) calculated in unidimensional logistic regression and adjusted (AOR) odds ratios calculated in multidimensional logistic regression: AOR-a was obtained after adjustment for maternal age and primiparity, and AOR-b was obtained after adjustment for age, primiparity and pre-pregnancy BMI; ** P-value <0.05 was statistically significant; *** Women without family history of hypertension. GH: gestational hypertension; PE: preeclampsia; BMI: body mass index; GWG: gestational weight gain; H: hypertension; GDM ; gestational diabetes mellitus; GDM-2: gestational diabetes treated with insulin.
