# Supplementary material for: Pre-Pregnancy Obesity vs. Other Risk Factors in Probability Models of Preeclampsia and Gestational Hypertension
Source: Nutrients. 2020 Sep 2;12(9):2681. doi: 10.3390/nu12092681 (PMC7551880; doi:10.3390/nu12092681)
Supplement: Supplementary file 1 [file nutrients-12-02681-s001.zip › Table S4.docx]

**Table S4.** Set of AUC values in the extended multivariate models in the assessment of the probability of gestational hypertension (GH) and preeclampsia (PE).

|  | **Gestational hypertension (GH)** | | | |  |  |
| --- | --- | --- | --- | --- | --- | --- |
|  | *0,539* | *Base model*  *0,421-0,656* | *0,520* | *Differences ** |  |  |
| **Extended models**  **(base model + listed variables)** | **AUC** | **± 95% CI** | **p **** | **AUC difference** | **± 95% CI** | **p **** |
| Pre-pregnancy BMI (kg/m²) | 0,716 | 0,663-0,77 | <0,001 | 0,116 | 0,064-0,17 | <0,001 |
| Pre-pregnancy BMI [c] | 0,704 | 0,65-0,758 | <0,001 | 0,104 | 0,053-0,156 | <0,001 |
| Pre-pregnancy weight (kg) | 0,697 | 0,644-0,751 | <0,001 | 0,097 | 0,047-0,149 | <0,001 |
| Pre-pregnancy BMI ≥ 25 kg/m² | 0,685 | 0,629-0,74 | <0,001 | 0,085 | 0,035-0,136 | 0,001 |
| Pre-pregnancy BMI ≥ 30 kg/m² | 0,663 | 0,606-0,721 | <0,001 | 0,063 | 0,023-0,105 | 0,002 |
| Prior GH/PE | 0,656 | 0,599-0,713 | <0,001 | 0,056 | 0,020-0,093 | 0,002 |
| GWG [c] | 0,653 | 0,595-0,710 | <0,001 | 0,053 | 0,007-0,099 | 0,024 |
| Financial status | 0,648 | 0,593-0,702 | <0,001 | 0,048 | 0,007-0,089 | 0,022 |
| No multivitamins | 0,646 | 0,592-0,7 | <0,001 | 0,046 | 0,002-0,09 | 0,039 |
| Smoking in I trimester | 0,646 | 0,591-0,701 | <0,001 | 0,046 | 0,006-0,087 | 0,025 |
| Education < 12 years | 0,643 | 0,590-0,696 | <0,001 | 0,043 | 0,007-0,080 | 0,018 |
| Family history; H in the mother[c] | 0,639 | 0,587-0,691 | <0,001 | 0,039 | 0,003-0,076 | 0,034 |
| Family history; H in the father [c] | 0,638 | 0,585-0,691 | <0,001 | 0,038 | 0,000-0,078 | 0,052 |
| No folic acid supplementation | 0,634 | 0,579-0,688 | <0,001 | 0,034 | 0,000-0,075 | 0,101 |
| Urogenital infection | 0,632 | 0,576-0,689 | <0,001 | 0,032 | 0,000-0,069 | 0,075 |
| Family history of Hypertension | 0,631 | 0,579-0,684 | <0,001 | 0,031 | 0,000-0,065 | 0,064 |
| Interpregnancy interval [c] | 0,619 | 0,564-0,675 | <0,001 | 0,019 | 0,014-0,048 | 0,166 |
| Hypothyroidism | 0,618 | 0,562-0,674 | <0,001 | 0,018 | 0,000-0,049 | 0,253 |
| GWG (kg) | 0,616 | 0,554-0,677 | <0,001 | 0,016 | 0,000-0,053 | 0,402 |
| Maternal height (cm) | 0,613 | 0,557-0,669 | <0,001 | 0,013 | 0,000-0,035 | 0,22 |
| Place of residence [c] | 0,611 | 0,557-0,665 | <0,001 | 0,011 | 0,000-0,034 | 0,318 |
| GWG > 15 kg | 0,607 | 0,549-0,664 | <0,001 | 0,007 | 0,000-0,03 | 0,551 |
| Treatment of infertility | 0,601 | 0,545-0,658 | <0,001 | 0,001 | 0,000-0,008 | 0,621 |
| In vitro fertilization | 0,6 | 0,543-0,656 | <0,001 | 0,000 | 0,000-0,005 | 0,925 |
| GWG > 10 kg | 0,598 | 0,542-0,655 | <0,001 | -0,002 | 0,000-0,006 | 0,599 |
|  | **Preeclampsia (PE)** | | | |  |  |
|  | *0,539* | *Base model*  *0,421-0,656* | *0,520* | *Differences ** |  |  |
| **Extended models**  **(base model + listed variables)** | **AUC** | **± 95% CI** | **p **** | **AUC difference** | **± 95% CI** | **p **** |
| Pre-pregnancy BMI [c] | 0,726 | 0,618-0,834 | <0,001 | 0,187 | 0,033-0,342 | 0,017 |
| Family history of H in the mother [c] | 0,717 | 0,631-0,803 | <0,001 | 0,178 | 0,044-0,312 | 0,009 |
| Pre-pregnancy BMI ≥ 30 kg/m² | 0,703 | 0,589-0,817 | 0,001 | 0,164 | 0,040-0,288 | 0,009 |
| Pre-pregnancy BMI ≥ 25 kg/m² | 0,679 | 0,576-0,782 | 0,003 | 0,140 | 0,005-0,276 | 0,042 |
| Pre-pregnancy BMI (kg/m²) | 0,678 | 0,554-0,801 | 0,003 | 0,139 | 0,000-0,308 | 0,107 |
| Family history of H in the father [c] | 0,658 | 0,554-0,762 | 0,008 | 0,119 | 0,000-0,259 | 0,094 |
| Financial status | 0,648 | 0,53-0,767 | 0,013 | 0,109 | 0,000-0,228 | 0,068 |
| Interpregnancy interval [c] | 0,646 | 0,542-0,75 | 0,015 | 0,107 | 0,000-0,25 | 0,138 |
| Pre-pregnancy weight (kg) | 0,642 | 0,516-0,768 | 0,017 | 0,103 | 0,000-0,274 | 0,233 |
| Family history of hypertension (H) | 0,638 | 0,536-0,739 | 0,021 | 0,099 | 0,000-0,224 | 0,119 |
| Education < 12 years | 0,629 | 0,505-0,753 | 0,031 | 0,090 | 0,000-0,181 | 0,05 |
| No folic acid supplementation | 0,625 | 0,503-0,746 | 0,037 | 0,086 | 0,007-0,165 | 0,032 |
| Prior GH/PE | 0,622 | 0,504-0,739 | 0,042 | 0,083 | 0,000-0,181 | 0,097 |
| Maternal height (cm) | 0,589 | 0,471-0,707 | 0,137 | 0,050 | 0,000-0,144 | 0,289 |
| Smoking in I trimester | 0,588 | 0,471-0,705 | 0,141 | 0,049 | 0,000-0,147 | 0,319 |
| GWG [c] | 0,586 | 0,467-0,706 | 0,149 | 0,047 | 0,000-0,161 | 0,407 |
| Place of residence [c] | 0,586 | 0,46-0,711 | 0,153 | 0,047 | 0,000-0,117 | 0,187 |
| Treatment of infertility | 0,585 | 0,463-0,707 | 0,157 | 0,046 | 0,000-0,111 | 0,163 |
| Urogenital infection | 0,568 | 0,448-0,689 | 0,255 | 0,029 | 0,000-0,074 | 0,188 |
| GWG (kg) | 0,565 | 0,429-0,7 | 0,28 | 0,026 | 0,000-0,169 | 0,72 |
| GWG > 15 kg | 0,563 | 0,45-0,676 | 0,29 | 0,024 | 0,000-0,098 | 0,507 |
| No multivitamins supplementation | 0,557 | 0,451-0,662 | 0,343 | 0,018 | 0,000-0,054 | 0,319 |
| In vitro fertilization | 0,555 | 0,434-0,675 | 0,360 | 0,016 | 0,000-0,04 | 0,174 |
| GWG > 10 kg | 0,555 | 0,438-0,673 | 0,355 | 0,016 | 0,000-0,065 | 0,49 |
| Hypothyroidism | 0,553 | 0,424-0,681 | 0,379 | 0,014 | 0,000-0,076 | 0,655 |

* Differences between extended models and base model; ** p<0.05 was assumed to be significant. AUC: area under receiver operating characteristic curve; [c]: categories of independent variables (are described in the Methodology); BMI: body mass index; GWG: gestational weight gain; H: hypertension in family history.
