# Supplementary material for: Pre-Pregnancy Obesity vs. Other Risk Factors in Probability Models of Preeclampsia and Gestational Hypertension
Source: Nutrients. 2020 Sep 2;12(9):2681. doi: 10.3390/nu12092681 (PMC7551880; doi:10.3390/nu12092681)
Supplement: Supplementary file 1 [file nutrients-12-02681-s001.zip › Table S5.docx]

**Table S5.** Set of values of Net Reclassification Improvement (NRI) in the extended multivariate models in the assessment of the probability of gestational hypertension (GH) and preeclampsia (PE).

| **Extended models**  **(base model + listed variables)** | **NRI (95% CI)** | **p *** | **NRI(1)** | **NRI(0)** | **ill = 1**  **down \| up** | **healthy = 0**  **down \| up** |
| --- | --- | --- | --- | --- | --- | --- |
|  |  | **GH** |  |  |  |  |
| Pre-pregnancy BMI [c] | 0.571 (0.378;0.765) | <0.001 | 8.0% | 49.2% | 46.02% \| 53.98% | 74.58% \| 25.42% |
| Pre-pregnancy BMI ≥ 25 kg/m² | 0.571 (0.378;0.765) | <0.001 | 8.0% | 49.2% | 46.02% \| 53.98% | 74.58% \| 25.42% |
| Pre-pregnancy weight (kg) | 0.544 (0.351;0.737) | <0.001 | 15.0% | 39.4% | 42.48% \| 57.52% | 69.68% \| 30.32% |
| Pre-pregnancy BMI (kg/m²) | 0.542 (0.348;0.736) | <0.001 | 11.5% | 42.7% | 44.25% \| 55.75% | 71.35% \| 28.65% |
| Prior GH/PE | 0.433 (0.247;0.62) | <0.001 | -23.9% | 67.2% | 61.95% \| 38.05% | 83.61% \| 16.39% |
| GWG [c] | 0.419 (0.223;0.614) | <0.001 | 9.7% | 32.1% | 45.13% \| 54.87% | 66.06% \| 33.94% |
| Pre-pregnancy BMI ≥ 30 kg/m² | 0.399 (0.23;0.568) | <0.001 | -45.1% | 85.0% | 72.57% \| 27.43% | 92.52% \| 7.48% |
| No multivitamins supplementation | 0.368 (0.174;0.563) | <0.001 | 16.8% | 20.0% | 41.59% \| 58.41% | 60% \| 40% |
| No folic acid supplementation | 0.312 (0.14;0.483) | <0.001 | 52.2% | -21.0% | 23.89% \| 76.11% | 39.48% \| 60.52% |
| Family history. H in the father [c] | 0.279 (0.086;0.472) | 0.005 | -15.0% | 43.0% | 57.52% \| 42.48% | 71.48% \| 28.52% |
| Lower financial status | 0.25 (0.085;0.416) | 0.003 | -50.4% | 75.5% | 75.22% \| 24.78% | 87.74% \| 12.26% |
| Family history. Hypertension (H) | 0.236 (0.04;0.432) | 0.019 | -4.4% | 28.0% | 52.21% \| 47.79% | 64% \| 36% |
| Family history. H in the mother [c] | 0.236 (0.04;0.432) | 0.019 | -4.4% | 28.0% | 52.21% \| 47.79% | 64% \| 36% |
| Urogenital infection | 0.22 (0.056;0.384) | 0.009 | -52.2% | 74.2% | 76.11% \| 23.89% | 87.1% \| 12.9% |
| Smoking in I trimester | 0.205 (0.07;0.341) | 0.003 | -69.9% | 90.5% | 84.96% \| 15.04% | 95.23% \| 4.77% |
| Hypothyroidism | 0.195 (0.035;0.355) | 0.017 | -55.8% | 75.2% | 77.88% \| 22.12% | 87.61% \| 12.39% |
| Education < 12 years | 0.159 (0.026;0.292) | 0.019 | -71.7% | 87.6% | 85.84% \| 14.16% | 93.81% \| 6.19% |
| GWG (kg) | 0.149 (-0.048;0.346) | 0.139 | 4.4% | 10.5% | 47.79% \| 52.21% | 55.23% \| 44.77% |
| Place of residence [c] | 0.144 (-0.024;0.312) | 0.094 | 54.0% | -39.6% | 23.01% \| 76.99% | 30.19% \| 69.81% |
| Maternal height (cm) | 0.125 (-0.072;0.322) | 0.213 | 6.2% | 6.3% | 46.9% \| 53.1% | 53.16% \| 46.84% |
| GWG > 15 kg | 0.113 (-0.079;0.305) | 0.248 | -22.1% | 33.4% | 61.06% \| 38.94% | 66.71% \| 33.29% |
| GWG > 10 kg | -0.011 (-0.191;0.168) | 0.903 | 41.6% | -42.7% | 29.2% \| 70.8% | 28.65% \| 71.35% |
| Interpregnancy interval [c] | -0.014 (-0.211;0.183) | 0.891 | 4.4% | -5.8% | 47.79% \| 52.21% | 47.1% \| 52.9% |
| Treatment of infertility | -0.074 (-0.241;0.093) | 0.386 | -54.0% | 46.6% | 76.99% \| 23.01% | 73.29% \| 26.71% |
| In vitro fertilization | -0.12 (-0.283;0.044) | 0.151 | -57.5% | 45.5% | 78.76% \| 21.24% | 72.77% \| 27.23% |
|  |  | **PE** |  |  |  |  |
| Pre-pregnancy BMI [c] | 0.688 (0.284;1.091) | 0.001 | 0.0% | 68.8% | 50% \| 50% | 84.39% \| 15.61% |
| Pre-pregnancy BMI ≥ 30 kg/m² | 0.6 (0.211;0.989) | 0.002 | -25.0% | 85.0% | 62.5% \| 37.5% | 92.52% \| 7.48% |
| Pre-pregnancy BMI ≥ 25 kg/m² | 0.575 (0.172;0.978) | 0.005 | 8.3% | 49.2% | 45.83% \| 54.17% | 74.58% \| 25.42% |
| Family history; H in the mother [c] | 0.563 (0.161;0.965) | 0.006 | -8.3% | 64.6% | 54.17% \| 45.83% | 82.32% \| 17.68% |
| Pre-pregnancy BMI (kg/m²) | 0.51 (0.107;0.914) | 0.013 | 8.3% | 42.7% | 45.83% \| 54.17% | 71.35% \| 28.65% |
| Family history. Hypertension (H) | 0.447 (0.046;0.847) | 0.029 | 16.7% | 28.0% | 41.67% \| 58.33% | 64% \| 36% |
| Family history. H in the father [c] | 0.447 (0.046;0.847) | 0.029 | 16.7% | 28.0% | 41.67% \| 58.33% | 64% \| 36% |
| Lower financial status | 0.422 (0.041;0.802) | 0.030 | -33.3% | 75.5% | 66.67% \| 33.33% | 87.74% \| 12.26% |
| Interpregnancy interval [c] | 0.405 (0.035;0.775) | 0.032 | 41.7% | -1.2% | 29.17% \| 70.83% | 49.42% \| 50.58% |
| Education < 12 years | 0.376 (0.028;0.724) | 0.034 | -50.0% | 87.6% | 75% \| 25% | 93.81% \| 6.19% |
| No folic acid supplementation | 0.373 (0.041;0.705) | 0.028 | 58.3% | -21.0% | 20.83% \| 79.17% | 39.48% \| 60.52% |
| Pre-pregnancy weight (kg) | 0.373 (-0.032;0.778) | 0.071 | 0.0% | 37.3% | 50% \| 50% | 68.65% \| 31.35% |
| Hypothyroidism | 0.252 (-0.097;0.602) | 0.157 | -50.0% | 75.2% | 75% \| 25% | 87.61% \| 12.39% |
| GWG [c] | 0.238 (-0.166;0.642) | 0.249 | -8.3% | 32.1% | 54.17% \| 45.83% | 66.06% \| 33.94% |
| Place of residence [c] | 0.174 (-0.157;0.506) | 0.302 | 58.3% | -40.9% | 20.83% \| 79.17% | 29.55% \| 70.45% |
| Maternal height (cm) | 0.157 (-0.248;0.562) | 0.448 | 8.3% | 7.4% | 45.83% \| 54.17% | 53.68% \| 46.32% |
| Smoking in I trimester | 0.155 (-0.112;0.421) | 0.255 | -75.0% | 90.5% | 87.5% \| 12.5% | 95.23% \| 4.77% |
| Prior GH/PE | 0.098 (-0.27;0.467) | 0.602 | -41.7% | 51.5% | 70.83% \| 29.17% | 75.74% \| 24.26% |
| GWG > 10 kg | 0.094 (-0.289;0.476) | 0.631 | -33.3% | 42.7% | 66.67% \| 33.33% | 71.35% \| 28.65% |
| Urogenital infection | 0.091 (-0.135;0.318) | 0.428 | 83.3% | -74.2% | 8.33% \| 91.67% | 12.9% \| 87.1% |
| GWG > 15 kg | 0.084 (-0.309;0.477) | 0.675 | -25.0% | 33.4% | 62.5% \| 37.5% | 66.71% \| 33.29% |
| GWG (kg) | 0.055 (-0.35;0.459) | 0.791 | -8.3% | 13.8% | 54.17% \| 45.83% | 56.9% \| 43.1% |
| No multivitamins supplementation | 0.05 (-0.343;0.443) | 0.803 | 25.0% | -20.0% | 37.5% \| 62.5% | 40% \| 60% |
| Treatment of infertility | -0.121 (-0.425;0.183) | 0.436 | -66.7% | 54.6% | 83.33% \| 16.67% | 77.29% \| 22.71% |
| In vitro fertilization | -0.183 (-0.487;0.122) | 0.239 | -66.7% | 48.4% | 83.33% \| 16.67% | 74.19% \| 25.81% |

* P-value <0.05 was statistically significant. NRI: Net Reclassification Improvement; NRI(1): NRI for ill group; NRI(0): NRI for healthy group; [c]: categories of independent variables (are described in the Methodology); BMI: body mass index; GWG: gestational weight gain; H: hypertension, in family history.

The comments:

Down: a decrease in the likelihood of disease.

Up: increase in the likelihood of disease.

The NRI (1) is an assessment for the sick participants, and it represents the difference in the value of the increase and the decrease in probability in the model after adding a factor. NRI (1) = 8% means that in 8% of ill patients there was a "correct" reclassification in the direction of increased disease probability after adding BMI.

The NRI (0) is an assessment for the healthy participants, and it represents the difference in the value of the increase and the decrease in probability in the model after adding a factor. NRI (0) = 49.2% means that in 49.2% of the healthy persons there was a "correct" reclassification towards a decrease in the probability of the disease after adding BMI.
