# Supplementary material for: Pre-Pregnancy Obesity vs. Other Risk Factors in Probability Models of Preeclampsia and Gestational Hypertension
Source: Nutrients. 2020 Sep 2;12(9):2681. doi: 10.3390/nu12092681 (PMC7551880; doi:10.3390/nu12092681)
Supplement: Supplementary file 1 [file nutrients-12-02681-s001.zip › Table S6.docx]

**Table S6.** Set of values of Integrated Discrimination Improvement (IDI) in the extended multivariate models in the assessment of the probability of gestational hypertension (GH) and preeclampsia (PE).

| **Extended models**  **(base model + listed variables)** | **IDI (95% CI)** | **P *** |
| --- | --- | --- |
|  | **GH** |  |
| Prior GH/PE | 0.068 (0.031;0.104) | <0.001 |
| Pre-pregnancy BMI (kg/m²) | 0.064 (0.041;0.087) | <0.001 |
| Pre-pregnancy BMI [c] | 0.058 (0.038;0.079) | <0.001 |
| Pre-pregnancy BMI ≥ 30 kg/m² | 0.045 (0.024;0.066) | <0.001 |
| Pre-pregnancy weight (kg) | 0.044 (0.026;0.063) | <0.001 |
| Pre-pregnancy BMI ≥ 25 kg/m² | 0.041 (0.026;0.057) | <0.001 |
| GWG [c] | 0.027 (0.015;0.038) | <0.001 |
| Smoking in I trimester | 0.024 (0.008;0.04) | 0.003 |
| No multivitamins supplementation | 0.016 (0.007;0.025) | 0.001 |
| Lower financial status | 0.015 (0.004;0.025) | 0.007 |
| No folic acid supplementation | 0.013 (0.006;0.02) | <0.001 |
| GWG (kg) | 0.011 (0.003;0.02) | 0.009 |
| Urogenital infection | 0.010 (0.001;0.019) | 0.022 |
| Education < 12 years | 0.010 (0.0003;0.019) | 0.043 |
| Hypothyroidism | 0.008 (0.001;0.015) | 0.032 |
| Family history. H in the father [c] | 0.008 (0.0001;0.016) | 0.046 |
| Family history. Hypertension (H) | 0.004 (-0.002;0.01) | 0.194 |
| Family history. H in the mother [c] | 0.004 (-0.002;0.011) | 0.190 |
| GWG > 15 kg | 0.004 (-0.0004;0.008) | 0.077 |
| Interpregnancy interval [c] | 0.004 (-0.001;0.009) | 0.086 |
| Maternal height (cm) | 0.003 (-0.001;0.007) | 0.137 |
| Place of residence [c] | 0.002 (-0.001;0.006) | 0.238 |
| Treatment of infertility | 0.001 (-0.002;0.003) | 0.562 |
| In vitro fertilization | 0.0005 (-0.001;0.002) | 0.600 |
| GWG > 10 kg | 0.0003 (-0.0005;0.001) | 0.454 |
|  | **PE** |  |
| Prior GH/PE | 0.05 (-0.006;0.105) | 0.080 |
| Pre-pregnancy BMI [c] | 0.034 (0.012;0.056) | 0.002 |
| Pre-pregnancy BMI ≥ 30 kg/m² | 0.032 (0.01;0.054) | 0.004 |
| Pre-pregnancy BMI (kg/m²) | 0.020 (0.003;0.037) | 0.019 |
| Education < 12 years | 0.018 (0.001;0.034) | 0.034 |
| Family history. H in the mother [c] | 0.013 (0.002;0.024) | 0.020 |
| Pre-pregnancy BMI ≥ 25 kg/m² | 0.012 (0.003;0.021) | 0.011 |
| Pre-pregnancy weight (kg) | 0.012 (0.0005;0.024) | 0.041 |
| Lower financial status | 0.012 (0.001;0.023) | 0.039 |
| Family history. H in the father [c] | 0.007 (-0;0003.014) | 0.059 |
| Interpregnancy interval [c] | 0.007 (-0.001;0.015) | 0.072 |
| GWG (kg) | 0.006 (-0.003;0.015) | 0.165 |
| Treatment of infertility | 0.005 (-0.003;0.013) | 0.251 |
| Hypothyroidism | 0.005 (-0.001;0.011) | 0.124 |
| Family history. Hypertension (H) | 0.005 (-0.0003;0.011) | 0.069 |
| No folic acid supplementation | 0.005 (0.001;0.009) | 0.010 |
| Smoking in I trimester | 0.004 (-0.003;0.011) | 0.266 |
| In vitro fertilization | 0.003 (-0.003;0.008) | 0.372 |
| Maternal height (cm) | 0.002 (-0.001;0.005) | 0.253 |
| GWG [c] | 0.002 (-0.001;0.006) | 0.205 |
| Place of residence [c] | 0.001 (-0.001;0.003) | 0.161 |
| Urogenital infection | 0.001 (-0.001;0.002) | 0.258 |
| GWG > 15 kg | 0.0004 (-0.001;0.002) | 0.577 |
| GWG > 10 kg | 0.0001 (-0.001;0.001) | 0.792 |
| No multivitamins supplementation | -0.0001 (-0.001;0.001) | 0.870 |

* P-value <0.05 was statistically significant. IDI: Integrated Discrimination Improvement; [c]: categories of independent variables (are described in the Methodology); BMI: body mass index; GWG: gestational weight gain; H: hypertension, in family history.
