# Supplementary material for: Pre-Pregnancy Obesity vs. Other Risk Factors in Probability Models of Preeclampsia and Gestational Hypertension
Source: Nutrients. 2020 Sep 2;12(9):2681. doi: 10.3390/nu12092681 (PMC7551880; doi:10.3390/nu12092681)
Supplement: Supplementary file 1 [file nutrients-12-02681-s001.zip › Figure S1.docx]

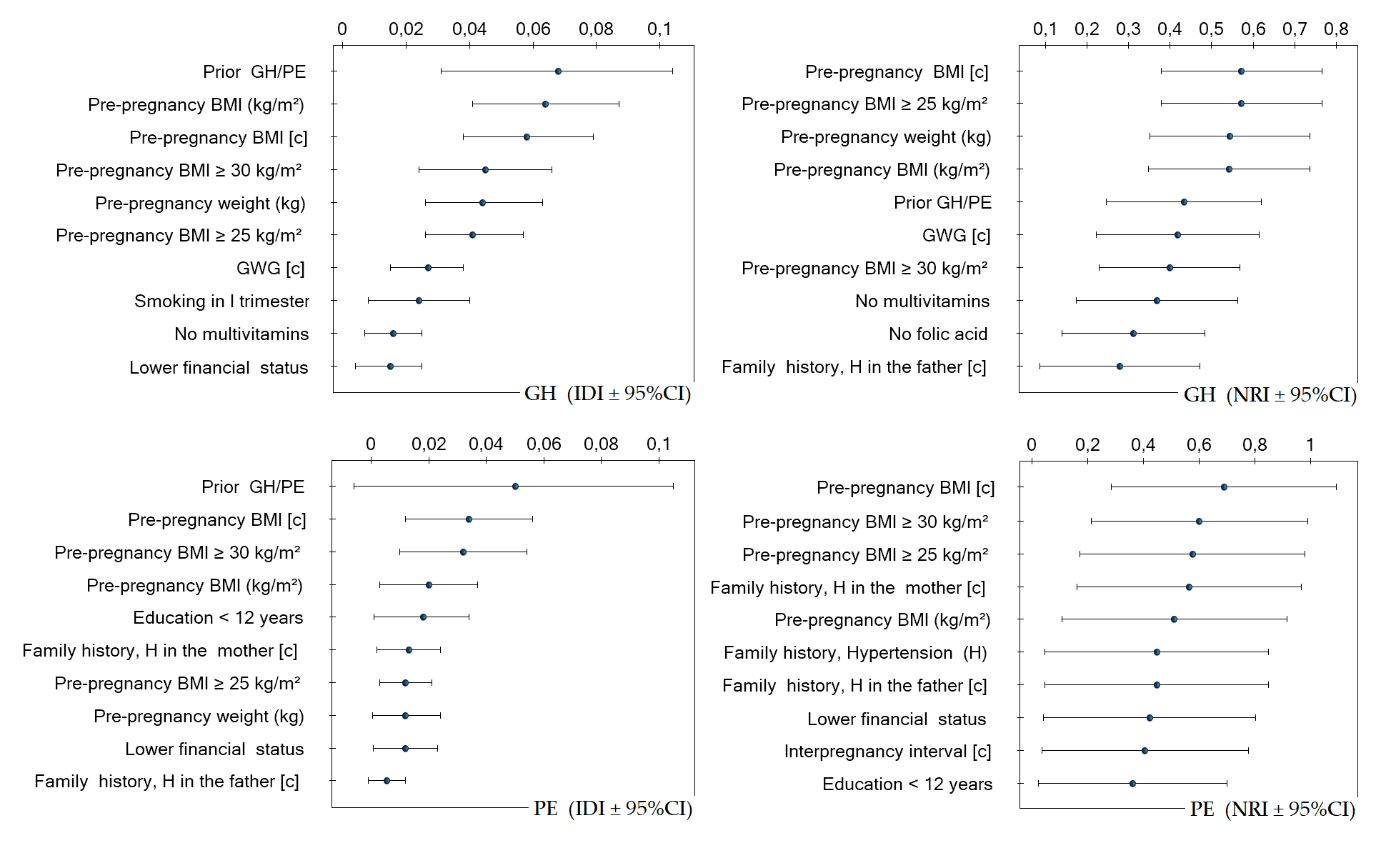


**Figure S1.** IDI and NRI index values after extending the predictive base model with the above-mentioned maternal characteristics. The graphs show the IDI and NRI values (black points) along with 95% confidence intervals (whiskers) after extending the base model (age + primiparity) with the maternal characteristics listed. [c]: categories as described for independent variables in the Methodology; IDI: Integrated Discrimination Improvement; NRI: Net Reclassification Improvement; BMI: body mass index; GWG: gestational weight gain; H: (chronic) hypertension in family history.
